# Common and Rare Genetic Risk Factors Converge in Protein Interaction Networks Underlying Schizophrenia

**DOI:** 10.3389/fgene.2018.00434

**Published:** 2018-09-28

**Authors:** Xiao Chang, Leandro de Araujo Lima, Yichuan Liu, Jin Li, Qingqin Li, Patrick M. A. Sleiman, Hakon Hakonarson

**Affiliations:** ^1^The Center for Applied Genomics, Children’s Hospital of Philadelphia, Philadelphia, PA, United States; ^2^Affiliated Cancer Hospital & Institute of Guangzhou Medical University, Guangzhou, China; ^3^Janssen Research & Development, LLC, Titusville, NJ, United States; ^4^Department of Pediatrics, The Perelman School of Medicine, University of Pennsylvania, Philadelphia, PA, United States; ^5^Division of Human Genetics, Children’s Hospital of Philadelphia, Philadelphia, PA, United States

**Keywords:** schizophrenia, GWAS, PPI Network, copy number variation (CNV), gene modules

## Abstract

Hundreds of genomic loci have been identified with the recent advances of schizophrenia in genome-wide association studies (GWAS) and sequencing studies. However, the functional interactions among those genes remain largely unknown. We developed a network-based approach to integrate multiple genetic risk factors, which lead to the discovery of new susceptibility genes and causal sub-networks, or pathways in schizophrenia. We identified significantly and consistently over-represented pathways in the largest schizophrenia GWA studies, which are highly relevant to synaptic plasticity, neural development and signaling transduction, such as long-term potentiation, neurotrophin signaling pathway, and the ERBB signaling pathway. We also demonstrated that genes targeted by common SNPs are more likely to interact with genes harboring *de novo* mutations (DNMs) in the protein-protein interaction (PPI) network, suggesting a mutual interplay of both common and rare variants in schizophrenia. We further developed an edge-based search algorithm to identify the top-ranked gene modules associated with schizophrenia risk. Our results suggest that the N-methyl-D-aspartate receptor (NMDAR) interactome may play a leading role in the pathology of schizophrenia, as it is highly targeted by multiple types of genetic risk factors.

## Introduction

Schizophrenia is a psychiatric disorder with profound genetic heterogeneity. Genetic risk factors of schizophrenia range in frequency from common to rare, including common single nucleotide polymorphisms (SNPs), recurrent rare copy number variants (CNVs) and *de novo* mutations (DNMs) (Friedman et al., 2008; International Schizophrenia Consortium, 2008; Vrijenhoek et al., 2008; Walsh et al., 2008; Xu et al., 2008; Glessner et al., 2010; Mulle et al., 2010; Girard et al., 2011; Levinson et al., 2011; Vacic et al., 2011; Kirov et al., 2012; Xu et al., 2012; Ripke et al., 2013; Sleiman et al., 2013; Fromer et al., 2014; Schizophrenia Working Group of the Psychiatric Genomics Consortium, 2014). Current genome-wide association studies (GWAS) in schizophrenia have reported 108 genome-wide significant loci, each of small effect size (Schizophrenia Working Group of the Psychiatric Genomics Consortium, 2014). It has also been reported that at least a quarter of the genetic contribution to schizophrenia risk can be explained by common SNPs (Lee et al., 2012; Ripke et al., 2013; Schizophrenia Working Group of the Psychiatric Genomics Consortium, 2014). On the other hand, multiple case-control studies have identified rare CNVs of strong effect to the risk of schizophrenia (International Schizophrenia Consortium, 2008; Vrijenhoek et al., 2008; Walsh et al., 2008; Xu et al., 2008; Glessner et al., 2010; Mulle et al., 2010; Levinson et al., 2011; Bergen et al., 2012; Kirov et al., 2012; Szatkiewicz et al., 2014). In addition, recent sequencing studies have shed new light on the genetic basis of schizophrenia that DNMs play a prominent part in the sporadic form of schizophrenia (Xu et al., 2012; Gulsuner et al., 2013; Fromer et al., 2014; McCarthy et al., 2014).

In these studies, multiple pieces of evidence show that genetic susceptibility of schizophrenia displays disruption across a group of functionally related genes implying a complex genetic network underlying schizophrenia (Glessner et al., 2010; Gulsuner et al., 2013; Fromer et al., 2014). To explore the network structure of schizophrenia, many network-based approaches have been applied to different types of genetic variations (Bullmore and Sporns, 2009; Gilman et al., 2012; Jia et al., 2012; Luo et al., 2014a,b). Among the different types of gene networks, protein-protein interaction (PPI) networks have been shown to be a powerful tool to identify the disease-associated modules and pathways, and reveal the biological significance of diverse genetic variations (Barabasi et al., 2011; Jia et al., 2011; Chang et al., 2013; Han et al., 2013; International Multiple Sclerosis Genetics Consortium, 2013; Leiserson et al., 2013; Luo et al., 2014b; Zhou et al., 2014). For example, instead of pursuing genome-wide significance, two GWA studies have successfully identified disease-associated gene modules, which are comprised of many closely interacting genes showing nominal significance, by integrating PPI networks analysis into GWAS (Han et al., 2013; International Multiple Sclerosis Genetics Consortium, 2013). However, it is still a challenge to conduct a comprehensive PPI network analysis, in particular by incorporating different types of genetic factors from different tissue types.

In the present study, we established a network-based approach to investigate the gene modules and pathways underlying schizophrenia, and to explore the inherent associations among multiple genetic risk factors. Our analysis uncovered significantly enriched association signals in pathways relevant to synaptic plasticity, neural development and signaling transduction such as long-term potentiation, neurotrophin signaling pathway, ERBB signaling pathway and MAPK signaling pathway, suggesting those play contributory roles in the pathophysiology of schizophrenia. We also demonstrated that genes targeted by common SNPs are more likely to interact with genes carrying DNMs. Finally, we identified a group of interacting genes showing a significant combined effect to the genetic susceptibility of schizophrenia.

## Materials and Methods

### GWAS Data Sets

Gene-level *P* values were calculated based on SNP *P* values from the largest GWAS conducted by Schizophrenia Psychiatric Genome-Wide Association Study Consortium (PGC), which recruited 36,989 cases and 113,075 controls (PGC phase 2, abbreviated as PGC2) (Schizophrenia Working Group of the Psychiatric Genomics Consortium, 2014). The association results were downloaded from the website of PGC^1^. As a control, we used the GWAS data of Crohn’s disease (CD) from the International IBD Genetics Consortium^2^ including a total of 3,685 cases and 5,968 controls (Jostins et al., 2012).

### Gene-Level Associations

Gene-level associations were calculated by VEGAS (Liu et al., 2010). VEGAS performs Monte-Carlo simulations from the multivariate normal distribution based on the LD pattern from reference populations and assigns an estimated *P* value to each gene. SNPs located within 50 kb upstream and 50 kb down stream of gene boundaries are used in the analysis in order to capture regulatory regions and SNPs in LD. Previous studies suggested *P*-value < 0.05 as the threshold of gene-level significance (Liu et al., 2010; International Multiple Sclerosis Genetics Consortium, 2013). However, since the number of genome-wide significant loci from the PGC2 study are much more than from the previous studies as a result of study size differences, the gene-level significance at both *P*-value < 0.01 (2501 significant genes) and *P*-value < 0.05 (4698 significant genes) was evaluated in this study. Genes located in the MHC region (25–34 mb on chr6) were excluded in the analysis.

### Rare Variations Curation

In this study, we used the sequencing results from previous studies (Xu et al., 2012; Gulsuner et al., 2013; Fromer et al., 2014) and annotated the variants by wANNOVAR^3^ (Chang and Wang, 2012). We used SIFT and Polyphen2 (HDIV) scores compiled by dbNSFP2 database as well as the AVSIFT score based on annotations at http://sift.bii.a-star.edu.sg to assess whether the missense variants are benign or damaging (**Supplementary Table S1**).

For the CNVs, we collected the genes disrupted by CNVs reported in large case-control studies of schizophrenia (**Supplementary Table S2**).

### Network Analysis

Schematic overview of the network analysis pipeline in this study was provided in **Supplementary Figure S2**.

The PPI Network was constructed based on the database iRefindex, which collected the protein interactions from a number of primary interaction databases (Razick et al., 2008). In order to control the rate of false positive interactions, we selected only those interactions that were supported by at least two independent PubMed literatures. A high-confidence network with 9,090 proteins (nodes) and 25,864 interactions (edges) was subsequently built for downstream analyses.

We next mapped the significant genes (*P* < 0.05) identified by VEGAS to the PPI network, and obtained a sub-network comprised of the significant genes and the interactions among them. The sub-network contains several connected components and many singletons. We then extracted the largest connected component (LCC) of the sub-network for downstream analysis.

**FIGURE 1:**
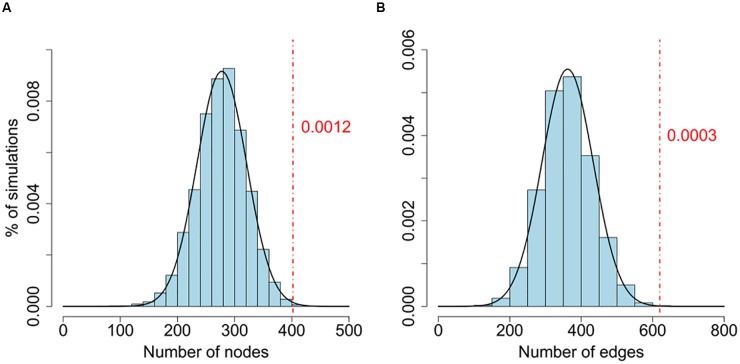
**(A,B)** Comparison of the number of nodes between the real network and random networks. Connectedness of the LCC based on gene-wise significant genes (*P*_gene_ < 0.01) from PGC2 study. The background distributions are generated by the number of nodes and edges of LCCs from 10,000 random simulations. *P* values are estimated by the proportion of LCCs from 10,000 random networks with more nodes or edges than the real network. Both node and edge numbers of the real data are significantly larger than random simulations (*P*_node_ = 0.0012; *P*_edge_ = 0.0003).

To test whether the size of the LCC is larger than what would be expected by chance, we randomly assigned *P* values of the same network and generated the simulated LCCs. We repeated this procedure 10,000 times, and use these simulations as background to estimate the significance of the LCCs generated from the real data (**Figure 1** and **Supplementary Table S3**). To investigate the biological significance of the genes in the LCC, we carried out a gene function enrichment analysis against the KEGG database using DAVID (**Supplementary Table S4**) (Huang et al., 2007).

### Gens (GWAS Edge-Based Network Search) Algorithm

Gens algorithm is modified based on a previously published node-based network search method (Ideker et al., 2002; Chuang et al., 2007; Jia et al., 2011).

Gens first assigns a weight to each edge of the network calculated by the gene-wise *P* values and mRNA expression correlations of interacting gene pairs (**Supplementary Data Sheet 1**). The weight of each edge is defined as

$$W_{\mathrm{ij}}=C_{\mathrm{ij}}\times \sqrt{P_{i}\times P_{j}}$$

where *C_ij_* denotes the Pearson Correlation Coefficient of interacting gene pairs, gene *i* and gene *j*. *P_i_* is the *P* value of Gene *i*, *P_j_* is the *P* value of Gene *j*.

The gene mRNA expression data were downloaded from Allen Brain Atlas^4^

The weight of each edge was then converted into a *Z* score

$$Z_{\mathrm{ij}}=\phi^{-1}(1-W_{\mathrm{ij}})$$

where ϕ^−1^ represents the inverse normal cumulative distribution function.

The score of gene module is defined as

$$Z_{m}=\sum Z_{\mathrm{ij}}/\sqrt{k}$$

where *k* is the number of edges in the module.

The search procedure starts from the seed edge, neighborhood interactors are added into the module if they can yield an increment greater than Z*_m_*×*r*, *r* is set to 0.05 in this study.

To evaluate the likelihood of the detected modules were identified by chance, Gens creates a background distribution by scoring 100,000 randomly generated modules with the same number of genes as the detected module. The significance is calculated as the proportion of those random generated modules whose Z*_m_* are larger than or equal to that of the identified module. Gens also adjusted the identified module size by defining a normalized module score

$$Z_{n}=(Z_{m}-\mathrm{mean}(Z_{m}(\pi )))/\mathrm{sd}(Z_{m}(\pi )),$$

where *Z_m_*(π) represents the distribution of Z*_m_* generated by 100,000 simulations.

## Results

### Enriched Pathways Underlying Schizophrenia

We first used VEGAS to convert the SNP associations into gene-level *P* values (**Supplementary Figure S1**). We next extracted the sub-networks by genes with a significant gene-level *P* value. The identified sub-networks are comprised of connected components and singletons. Among the connected components, the LCC contains most of the nodes and edges in the sub-network, which may participate in potential pathways underlying schizophrenia. To investigate the biological significance of the LCCs, we carried out a gene function enrichment analysis on the gene set of LCCs. We found significantly over-represented KEGG pathways, which are highly relevant to synaptic plasticity, neural development and signaling transduction such as long-term potentiation, neurotrophin signaling pathway, ERBB signaling pathway, MAPK signaling pathway, and T cell receptor signaling pathway. Other enriched pathways include proteasome, ubiquitin mediated proteolysis pathway and multiple cancers associated pathways (**Supplementary Table S4**).

We further confirmed that the sizes of LCCs are significantly larger than the LCCs generated by simulated random networks (**Figure 1** and **Supplementary Table S3**). For comparison, we performed the same analysis on a CD cohort, the LCC size is also larger than random simulations (**Supplementary Table S3**). This result is consistent with a previous study pointing to a biological plausibility that a set of genes coherently contribute to disease risk through interactive co-function and co-regulation (International Multiple Sclerosis Genetics Consortium, 2013).

### Mutual Interplay of Common and Rare Genetic Risk Factors in Schizophrenia

**FIGURE 2:**
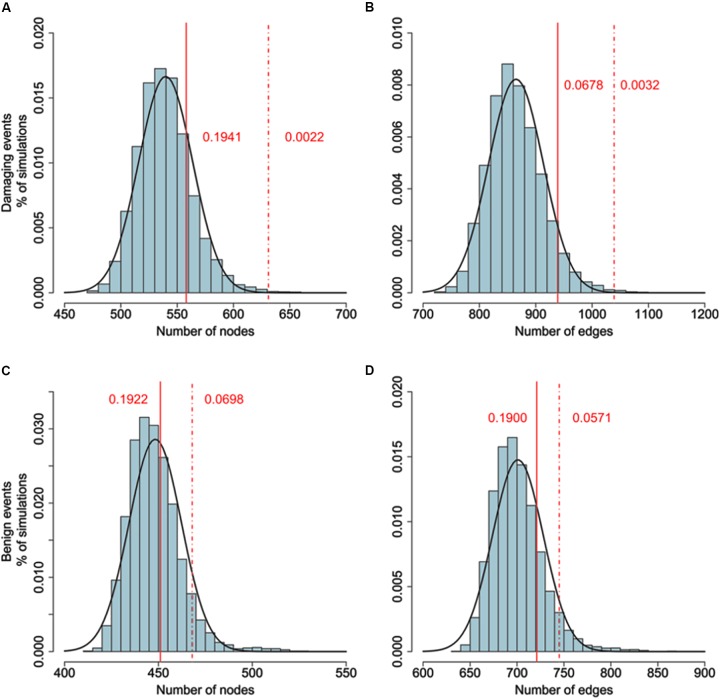
**(A,B)** Comparison of the number of nodes between the real network (damaging events) and random networks. **(C,D)** Comparison of the number of nodes between the real network (benign events) and random networks. Connectedness of the LCC based on gene-level significant genes (*P*_gene_ < 0.01) from PGC2 study and genes harboring DNMs. Original size of LCC based on gene-wise significant genes constituting 402 nodes and 620 edges. 635 genes harboring DNMs are added to generate the new LCC. The background distribution is generated by 10,000 LCCs based on adding 635 random selected genes. *P* values are estimated by the proportion of LCCs from random networks with more nodes or edges than the real network. As a control, we use the LCC generated by adding top 635 gene-level significant genes from Crohn’s disease as control. Dash line denotes the size of LCC generated by adding DNMs. Solid line denotes the size of LCC generated by adding CD top genes. Adding DNMs significantly increased the size of LCCs (DNMs: *P*_node_ = 0.0022, *P*_edge_ = 0.0032; CD: *P*_node_ = 0.1941, *P*_edge_ = 0.0678), while adding top CD genes did not. For comparison, we also added the synonymous and non-frameshift substitutions to generate the new LCC. The size of new LCC is not significantly larger than random simulations (Benign substitutions: *P*_node_ = 0.698, *P*_edge_ = 0.0571; CD: *P*_node_ = 0.1922, *P*_edge_ = 0.1900).

To examine whether genes belonging to the LCC network and identified by GWAS data are more likely to interact with genes harboring DNMs, We added the genes carrying potential DNMs (frameshift insertions/deletions, missense variants, or nonsense variants) and extracted the LCC based on the merged gene set. The size of the LCC significantly increased, larger than 10,000 simulations of the above procedure based on the same number of randomly selected genes. As a control, we tested the same number of top significant genes from CD GWAS. The size of the resulting LCC was not significantly different from random simulations. Furthermore, we also found the size of LCC did not increase significantly than random simulations if genes with silent *de novo* variants in schizophrenia cases were included (**Figure 2** and **Supplementary Table S5**).

### Causal Gene Modules Identified by Network Search Algorithm

In an attempt to add some more understanding to the schizophrenia genetic puzzle, we collected evidence for literature reported genes that are known to be disrupted by CNVs in schizophrenia patients (**Supplementary Table S2**), and added them to the PPI network analysis. We subsequently derived the LCC from genes targeted by SNPs, DNMs, and CNVs.

To pinpoint a small group of interactive genes with significant combined/additive effect to schizophrenia, we developed an edge-based network search algorithm (Gens) for detecting causal gene modules in PPI networks (**Supplementary Figure S2**). The results from gene-level significance at both 0.05 and 0.01 were highly consistent with each other demonstrating that the top-ranked gene modules overlapped considerably in their gene content. The shared genes between top-ranked modules significantly pointed to the interactome of N-methyl-D-aspartate receptor (NMDAR) genes including *DLG1*, *DLG2*, *DLG4*, *ERBB4*, *GRIN2A,* and *GRIN2B* (**Supplementary Figure S3**). All of those genes exhibited strong associations with schizophrenia susceptibility (*DLG1*, rs436564, *P* = 8.97 × 10^−4^; *DLG2*, rs12294291, *P* = 4.90 × 10^−7^; *DLG4*, rs222854, *P* = 3.76 × 10^−5^; *ERBB4*, rs16846200, *P* = 1.62 × 10^−5^; *GRIN2A*, rs9922678, *P* = 6.72 × 10^−9^; *GRIN2B*, rs11757887, *P* = 8.81 × 10^−7^; **Supplementary Figure S4**) with *GRIN2A,* reaching genome-wide significance in the PGC2 study.

**FIGURE 3:**
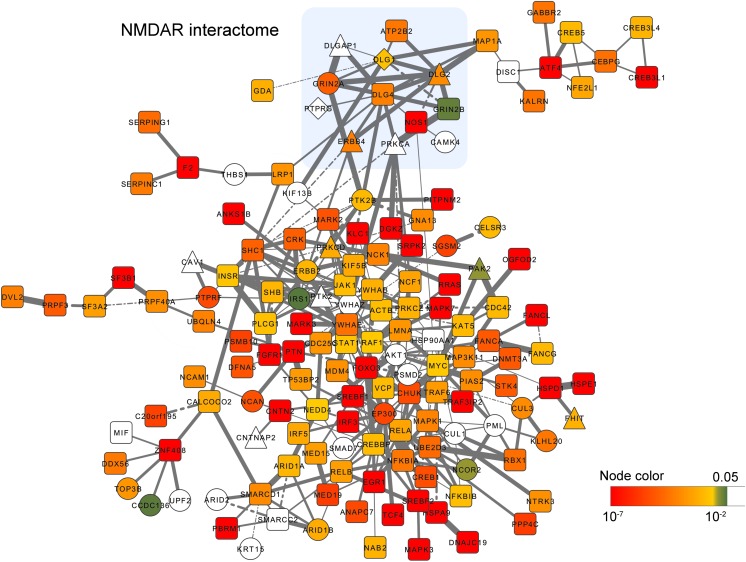
PPI network visualization of the most significant gene module derived from the network analysis. Gene-level *P* values (<0.05) are colored from green to red. Genes harboring DNMs and CNVs are shown as circles, and triangles respectively. Genes harboring both DNMs and CNVs are diamond shaped. Edges width reflects the gene co-expression correlation between two connected nodes. Solid and dash line denote positive, and negative correlations respectively.

Some of the NMDAR genes are also targeted by rare variations. For example, *DLG1* and *GRIN2A* have been reported to be targeted by DNMs; *DLG1*, *DLG2,* and *ERBB4* have been reported to be targeted by CNVs. To further explore the risk genes from the PPI network, we next select all the gene modules with *P* < 0.05 (*P* value calculated by random simulation, see Methods) and calculated the frequency of genes occurring in the selected modules. Genes with the frequency above the upper quartiles were defined as ‘top genes’. The ‘top genes’ was used to construct a new PPI network of 152 nodes and 324 edges (**Figure 3**), which reflects the most significant gene module derived from the network analysis.

Enrichment analysis indicated that they are enriched in the neurotrophin signaling pathway (*P* = 7.27 × 10^−13^), ERBB signaling pathway (*P* = 1.84 × 10^−7^), long-term potentiation (*P* = 5.37 × 10^−5^), MAPK signaling pathway (*P* = 3.16 × 10^−5^), T cell receptor signaling pathway (*P* = 1.17 × 10^−5^), and pathways in cancer (*P* = 4.87 × 10^−8^) to name a few (**Supplementary Table S6**). Moreover, in this network, we found multiple genes are connected with the core members of NMDAR interactome, such as *ATP2B2*, *DLGAP*, *MAP1A*, *NOS1*, *PTK2B*, *PTPRG* and *PRKCA*. Among them, *ATP2B2* (rs9879311, *P* = 2.77 × 10^−6^) and *NOS1* (rs2293052, *P* = 1.24 × 10^−6^) exhibited strong associations with schizophrenia risk in the PGC2 GWAS.

Beside the NMDAR interactome, we also found candidate genes showing strong associations with schizophrenia risk in the network, such as *ANKS1B* (rs10745841, *P* = 1.28 × 10^−6^), *CHUK* (rs975752, *P* = 2.52 × 10^−6^), *CNTN2* (rs16937, *P* = 8.69 × 10^−7^), *CNTNAP2* (rs6961013, *P* = 4.80 × 10^−5^), *CREB1* (rs2709410, *P* = 4.07 × 10^−6^), *CREB5* (rs4722797, *P* = 7.58 × 10^−6^; rs887622, *P* = 8.79 × 10^−6^), *CUL3* (rs11685299, *P* = 1.11 × 10^−8^), *EP300* (rs9607782, *P* = 6.76 × 10^−12^), *GABBR2* (rs2304389, *P* = 3.81 × 10^−7^), *GNA13* (rs11868185, *P* = 4.44 × 10^−5^), *NCOR2* (rs2229840, *P* = 2.90 × 10^−4^), *NTRK3* (rs146797905, *P* = 3.35 × 10^−7^; rs8042993, *P* = 7.84 × 10^−6^), *PAK2* (rs10446497, *P* = 5.30 × 10^−6^), *PTK2* (rs4961278, *P* = 1.86 × 10^−5^), *PTK2B* (rs2565065, *P* = 1.94 × 10^−7^), *PTN* (rs3735025, *P* = 7.75 × 10^−9^), *PTPRF* (rs11210892, *P* = 4.97 × 10^−10^), *STK4* (rs6065777, *P* = 5.92 × 10^−6^), *TCF4* (rs9636107, *P* = 9.09 × 10^−13^). Among them, *CUL3*, *EP300*, *NCOR2*, *PTK2B,* and *PTPRF* were targeted by DNMs, and *PAK2*, *PARK2* and *PTK2* were targeted by CNVs.

## Discussion

Given the heterogeneity and complexity of the genomic landscape in schizophrenia, we employed multiple network-based methods to reveal the instinct associations among different types of genetic risk variants, resulting in the discovery of novel gene modules and pathways underlying schizophrenia (**Supplementary Figure S2**).

With the recent GWAS success measures in schizophrenia uncovering 108 genome-wide significant loci (Schizophrenia Working Group of the Psychiatric Genomics Consortium, 2014), the genetic underpinning of this complex disease have begun to unravel. However, a considerable number of nominally significant loci are likely to be identified in future studies through the analysis of larger sample sizes or the application of new and innovative methods. For example, the schizophrenia susceptibility gene *CAMKK2* showing nominal significance (rs1063843, *P* = 2.32 × 10^−5^) in the PGC2 study was successfully identified by integrative analysis of gene expression and PPI (Luo X.J. et al., 2014).

We hypothesize that a group of functionally related genes with nominal significance could jointly contribute to schizophrenia susceptibility. We further performed a PPI network-based pathway analysis on two GWA studies of schizophrenia and identified significantly enriched KEGG pathways in both studies. Some pathways have been strongly associated with schizophrenia, such as the long-term potentiation, ERBB signaling pathway and MAPK signaling pathway (Fazzari et al., 2010; Pitcher et al., 2011; Funk et al., 2012; Savanthrapadian et al., 2013; Salavati et al., 2015). Interestingly, we found both the proteasome pathway and the ubiquitin mediated proteolysis pathway to be significantly enriched (**Supplementary Table S4**). Dysfunction of the ubiquitin-proteasome pathway (UPP) has been implicated in the pathology of various neurodegenerative conditions, and has been linked to several late-onset neurodegenerative diseases caused by aggregate-prone proteins such as Alzheimer’s disease Parkinson’s disease and Huntington’s disease (Rubinsztein, 2006; Hegde and Upadhya, 2011). Cumulative evidence also suggests that schizophrenia patients have aberrant gene expression patterns and protein expression disruptions in the UPP suggesting the UPP may also contribute to the deficits in schizophrenia (Vawter et al., 2001; Aston et al., 2004; Altar et al., 2005; Bousman et al., 2010; Rubio et al., 2013). Our results are consistent with these findings and provide new evidence in support of the association between the UPP and the pathogenesis of schizophrenia.

Cumulative evidence suggests that DNMs are an important cause of mental disorders such as schizophrenia, autism and intellectual disability (Veltman and Brunner, 2012). DNMs occur in different genes of different patients may be collectively responsible for a portion of sporadic schizophrenia cases. However, unlike CNVs, genes recurrently mutated by SNVs are rare and the overlap of genes disrupted by DNMs from recent studies is also small (**Supplementary Figure S5**). Thus, we naturally raise the question if genes targeted by common SNPs are more likely to be targeted by DNMs, and if genes targeted by common SNPs are more likely to interact with genes carrying DNMs? For the first question, the PGC2 study unveiled significant overlap between genes in the schizophrenia GWAS associated intervals and those with DNMs in schizophrenia (*P* = 0.0061) (Schizophrenia Working Group of the Psychiatric Genomics Consortium, 2014). For the second question, our analysis provides new evidence suggesting that genes targeted by common SNPs or DNMs are likely to interact with each other or participant in the same pathway. Collectively, these results suggest that schizophrenia susceptibility involves a mutual interplay of both common and rare genetic risk factors.

We additionally developed an edge-based network search algorithm to identify the leading disease associated modules underlying schizophrenia. The network search method was initially node-based, and developed in order to detect a group of interactive genes which show significantly changes in mRNA expression (Ideker et al., 2002). Later, this method was successfully applied on the post-GWAS network analysis (Jia et al., 2011; Jia et al., 2012; Han et al., 2013; International Multiple Sclerosis Genetics Consortium, 2013). Here, the advantage of Gens is that the edge-based method can utilize not only the node *P* values for the node but also the gene co-expression information as edge weights to score and rank the detected modules (**Methods**).

Using this approach, we found the top-ranked modules were significantly enriched in the NMDAR pathway associated genes including *DLG1*, *DLG2*, *DLG4*, *ERBB4*, *GRIN2A*, and *GRIN2B*. All of those genes show strong association with schizophrenia from GWAS. *DLG1*, *DLG2*, *ERBB4,* and *GRIN2A* were also targeted by DNMs or CNVs. In addition to *GRIN2A*, which has surpassed genome-wide significance (rs9922678, *P* = 6.72 × 10^−9^) in the PGC2 study, *DLG2* (rs12294291, *P* = 4.90 × 10^−7^), *GRIN2B* (rs11757887, *P* = 8.81 × 10^−7^) also showed strong associations nearly reaching genome-wide significance. These results suggested that the dysfunction of the NMDAR complex plays a leading role in the pathology of schizophrenia and is highly impacted by multiple genetic risk factors.

We further pinpointed two genes *ATP2B2* (rs9879311, *P* = 2.77 × 10^−6^) and *NOS1* (rs2293052, *P* = 1.24 × 10^−6^), which were closely connected to the NMDAR interactome and showed strong associations with schizophrenia risk. *ATP2B2* encodes the plasma membrane calcium-transporting ATPase 2 which plays an important role in intracellular calcium homeostasis and extrudes Ca^2+^ from cytosol into extracellular space. Family-based association studies suggested *ATP2B2* as a risk gene for autism in multiple ethnicities (Carayol et al., 2011; Prandini et al., 2012; Yang et al., 2013). A previous study also suggested *ATP2B2* could confer risk to schizophrenia (Ikeda et al., 2010). *NOS1* encodes a member of nitric oxide synthases, which functions as a biologic mediator in neurotransmission. Previous studies also provided evidence of the associations between *NOS1* and schizophrenia risk (Shinkai et al., 2002; Reif et al., 2011; Zhang et al., 2014).

Besides the NMDAR interactome, *CUL3*, *EP300*, *PTN*, *PTPRF*, *TCF4* reached genome-wide significance in the PGC2 study. *CUL3*, *EP300,* and *PTPRF* were also targeted by DNMs. *EP300* servers as an important hub in the network which directly interacted with 14 genes (*TCF4*, *EGR1*, *SREBF1,* and *SREBF2* located in genome-wide significant regions; *AKT1* and *SMAD7* targeted by DNMs). The product of *EP300* functions as histone acetyltransferase and regulates transcription via chromatin remodeling. Defects of *EP300* can cause Rubinstein-Taybi syndrome (a disease with short stature and intellectual disability) and may result in the formation of tumors (Tillinghast et al., 2003; Roelfsema et al., 2005; Negri et al., 2015). Interestingly, the DNM (NM_001429, exon14, c.C2656G, p.P886A) found in *EP300* is not predicted as damaging by either SIFT nor PolyPhen2, and a common missense variant in *EP300* is also strongly associated with schizophrenia (rs20551, *P* = 1.38 × 10^−8^; NM_001429, exon15, c.A2989G, p.I997V), which suggest that slight changes in the protein conformation of *EP300* may confer risk to schizophrenia. *EP300* is also interacted and co-expressed with *CREB1* in the network. It is reported that *EP300* can mediate cAMP-gene regulation through phosphorylated CREB proteins. *CREB1* also showed strong association (rs2709410, *P* = 4.07 × 10^−6^) in the PGC2 study. *CREB1* has been linked to drug addiction, memory disorders and neurodegenerative diseases (Bilecki and Przewlocki, 2000; Nestler, 2002; Josselyn and Nguyen, 2005; Lee et al., 2005). There is also some evidence of the association between *CREB1* and schizophrenia (Li et al., 2013; Ma et al., 2014; Kumar et al., 2015). *PTN* is another important hub, which interacted with eight genes (*NCAN*, *PSMB10,* and *SGSM2* located in genome-wide significant regions; *NCAN*, *PSMD2,* and *SGSM2* targeted by DNMs). *PTN* encodes pleiotrophin, which may suppress long-term potentiation induction (Pavlov et al., 2002).

In the network, candidate genes with nominal significance such as *ANKS1B*, *CNTN2*, *CNTNAP2*, *GABBR2*, *NCOR2,* and *NTRK3* also may be involved in the pathology of schizophrenia. The product of *ANKS1B* is predominantly expressed in brain tissue and interacted with amyloid beta protein precursor, which may play a role in brain development. A recent study demonstrated that *ANKS1B* product regulates synaptic GluN2B levels and further influence the NMDAR function. Multiple pieces of evidence have linked *CNTN2*, *CNTNAP2*, *GABBR2*, and *NTRK3* to neuropsychiatric disorders, including schizophrenia (Weickert et al., 2005; Friedman et al., 2008; Otnaess et al., 2009; Fazzari et al., 2010; Fatemi et al., 2011; Roussos et al., 2012; Bormuth et al., 2013; Fatemi et al., 2013; Karayannis et al., 2014). SNPs in *NCOR2* are associated with cocaine dependence in a recent GWAS (Gelernter et al., 2014).

In conclusion, the heterogeneity and complexity of the genetic landscape in schizophrenia is high. Here, we demonstrate that common and rare genetic risk factors converge on PPI networks that are enriched for schizophrenia candidate genes involved in synaptic plasticity and neural development. We also provide new evidence demonstrating that the NMDAR interactome is highly targeted by multiple types of genetic risk factors and may play a leading role in the risk of schizophrenia. Furthermore, we pinpointed many nominally significant genes in GWAS showing strong evidence to influence schizophrenia risk according to their network properties. These genes may reach genome-wide significance or carry DNMs to be unveiled in further genetic studies with more samples.

## Author Contributions

XC and HH designed the research. XC, LL, YL, and JL performed the analysis. QL and PS provided guidance for the analysis. XC and HH wrote and finalized the paper.

## Conflict of Interest Statement

QL was employed by company Janssen Research & Development, LCC. The remaining authors declare that the research was conducted in the absence of any commercial or financial relationships that could be construed as a potential conflict of interest.

## Footnotes

1 http://www.med.unc.edu/pgc/downloads

2 http://www.ibdgenetics.org

3 http://wannovar.usc.edu

4 http://human.brain-map.org/static/download
