# Supplementary material for: Common and Rare Genetic Risk Factors Converge in Protein Interaction Networks Underlying Schizophrenia
Source: Front Genet. 2018 Sep 28;9:434. doi: 10.3389/fgene.2018.00434 (PMC6172705; doi:10.3389/fgene.2018.00434)
Supplement: Supplementary file 7 [file Data_Sheet_1.PDF]

Supplementary Note

Determination of the edge weight by Gens

To combine the SNP *P* values with their physical distance, Qiao et al defined a distance measure for their massive multi-locus association test[1]. In this study, we used a similar equation to combine the gene *P* values with gene expression correlations,

$$W_{ij} = C_{ij} \times \sqrt{P_i \times P_j}$$

where C_{ij} denotes the Pearson Correlation Coefficient of interacting gene pairs, gene *i* and gene *j*. P_i is the *P* value of Gene *i*, P_j is the *P* value of Gene *j*.

Initially, gene *P* values are the gene-wise *P* values calculated by VEGAS. If the gene is targeted by DNMs, the gene *P* value will be multiplied by 0.01. If the gene is targeted by CNVs, the gene *P* value will be multiplied by 10^{-5}.

The gene expression correlations were calculated based on the mRNA microarray data from Allan Brain Atlas, which collected the gene expression value from more than 200 brain regions of 6 subjects (http://human.brain-map.org/static/download).

Gens search procedure

Gens perform a greedy search based on each edge in the network as a seed iteratively[2,3]. The procedure is as follow:

1.Initially, the module is seeded with only one edge. The score of seed module is calculated by the definition of Z_m.

2.Neighbors of any nodes in the seed module will be added to the seed iteratively. Neighbors of a given node are nodes whose network distances from the give node are less than d ($d = 2$).

3.The neighbors generating the maximum increment of Z_m will be added to the seed module if the increment is greater than $Z_m \times r$ ($r = 0.05$).

4.The iteration process will be stopped if none of neighbors can generate an increment greater than $Z_m \times r$.

The values of parameters d and r used in the search procedure were adopted from a previous study to avoid overfitting and control marginal effect[2].

Table S1. DNMs identified by three sequencing studies of schizophrenia (included as a separated excel file).

Table S2. Schizophrenia susceptibility genes from literature (Genes disrupted by CNVs in schizophrenia patients and three schizophrenia susceptibility genes).

**Gene**	**CNV**	**Reference**
DISC1	No	[4]
NRG1	No	[5]
DLG4	No	[6,7]
ERBB4	Yes	[8]
NRXN1	Yes	[8-11]
MYT1L	Yes	[10]
ASTN2	Yes	[10]
CTNND2	Yes	[10]
SLC1A3	Yes	[8]
DLG2	Yes	[8,12,13]
PRKCD	Yes	[8]
PRKAG2	Yes	[8]
PTK2	Yes	[8]
CAV1	Yes	[8]
PTPRM	Yes	[8]
LAMA1	Yes	[8]
MAGI2	Yes	[8]
GRMT	Yes	[8]
CNTNAP2	Yes	[9,14]
VIPR2	Yes	[11,15]
DLG1	Yes	[11,12,16]
PAK2	Yes	[11,16]
EHMT1	Yes	[12]
DLGAP1	Yes	[12]
CACNA1B	Yes	[17]
DOC2A	Yes	[17]
RET	Yes	[17]

RIT2	Yes	[17]
BARD1	Yes	[13]
FHIT	Yes	[13]
LRP1B	Yes	[13]
PRKCA	Yes	[13]
CIT	Yes	[13]
RAPGEF6	Yes	[13]
PTPRG	Yes	[17]
CAMK2D	Yes	[17]
PARK2	Yes	[17]
NEDD4L	Yes	[11]

Table S3. Statistics of LCCs generated by genes with significant gene-wise *P* values.

	node	P_{node}	edge	P_{edge}
PGC2 ($P_{gene}<0.05$)	1114	<0.0001	2012	<0.0001
PGC2 ($P_{gene}<0.01$)	402	0.0012	620	0.0003
CD ($P_{gene}<0.05$)	104	0.0462	136	0.0242

Table S4. KEGG pathway enrichment analysis of the LCCs by DAVID (included as a separated excel file).

Table S5. Statistics of LCCs generated by adding genes harboring DNMs or *de novo* silent substitutions in schizophrenia. (included as a separated excel file).

Note: Some neuronal signaling associated genes were found carrying *de novo* silent mutations in the study of Fromer et al, such as *HTT*, *NOTCH1*, and *NRXN2*. This

may result in inflation of the results. For example, the P_{node} and P_{edge} of LCCs generated by adding genes harboring *de novo* silent substitutions were < 0.05 (P_{node} < 0.0137, P_{edge} < 0.0176) for the gene-level significance at P_{gene} < 0.05. However, comparing to the P_{node} and P_{edge} of LCCs generated by adding genes harboring *de novo* mutations (P_{node} < 0.0002, P_{edge} < 0.0012), it is still much greater. Moreover, if we only use the data from Xu et al and Gulsuner et al, it is not significant.

Table S6. KEGG pathway enrichment analysis of the top gene modules by DAVID.

Figure S1. Manhattan plot of SNP level associations (upper panel) and gene level associations (lower panel). Red dash line denotes the genome- wide significant line (5×10^{-8} for SNP level and 2.8×10^{-6} for gene level). Blue dash line denotes the nominal significant line (10^{-5} for SNP level and 0.05 for gene level). The SNP level and gene level associations are basically specular.

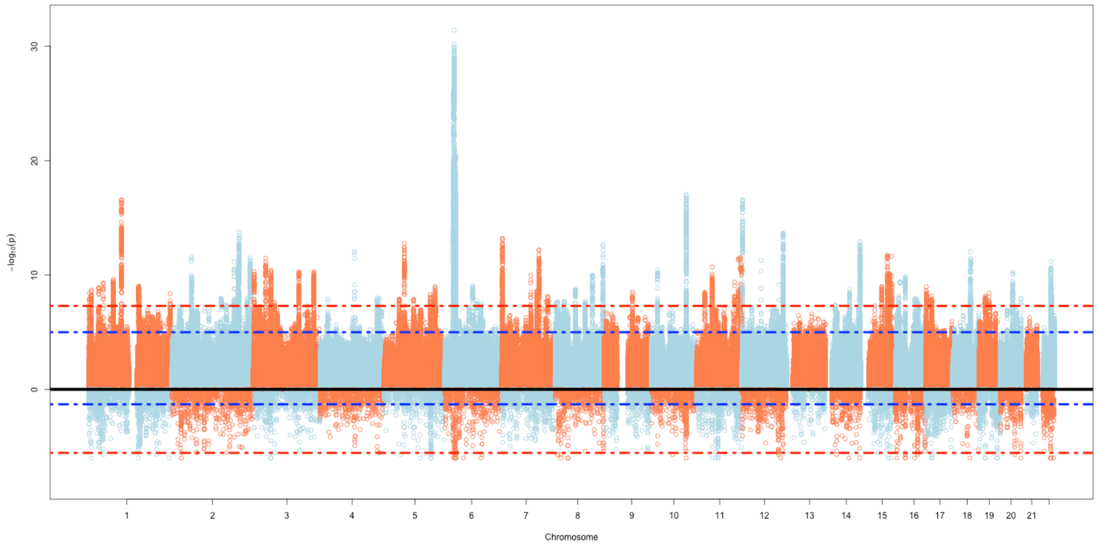

Figure S2. Schematic overview of the network analysis in this study

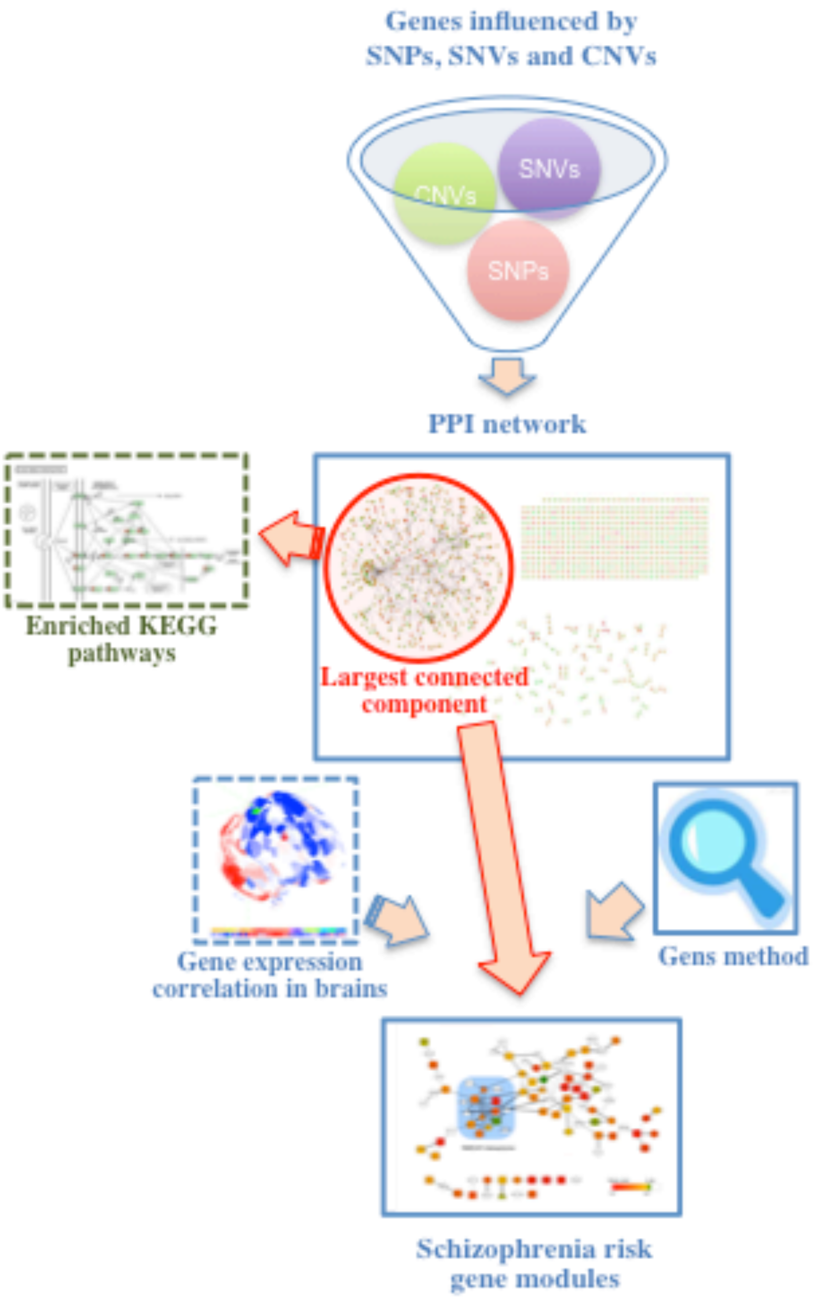

Figure S3 PPI network visualization of the top three significant modules identified by Gens. Gene-level *P* values (<0.05) are colored from green to red. Genes harboring *de novo* damaging SNVs and CNVs are shown as circles and diamonds respectively. Genes harboring both DNMs and CNVs are diamond shaped. Edges width reflects the gene co-expression correlation between two connected nodes. Solid and dash line denote positive and negative correlations respectively.

1. Top three significant modules identified when gene-level significance was set at $P_{node} < 0.01$

Module 1 (seed: ATP2B2_DLG1)

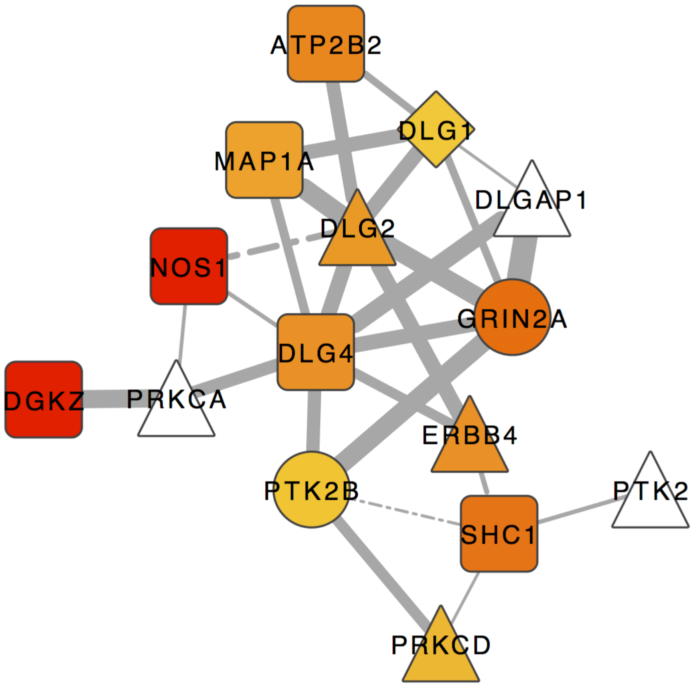

Module 2 (seed: DLG1_SEMA4C)

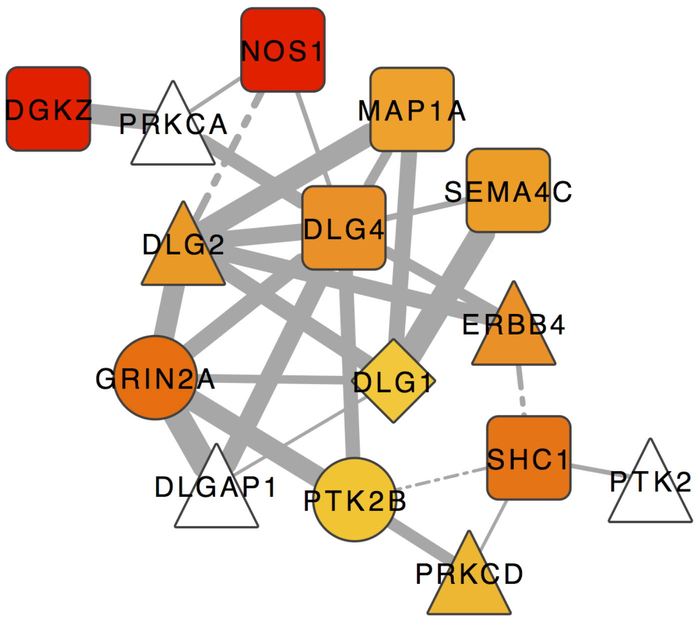

Module 3 (seed: DLG4_PTPRG)

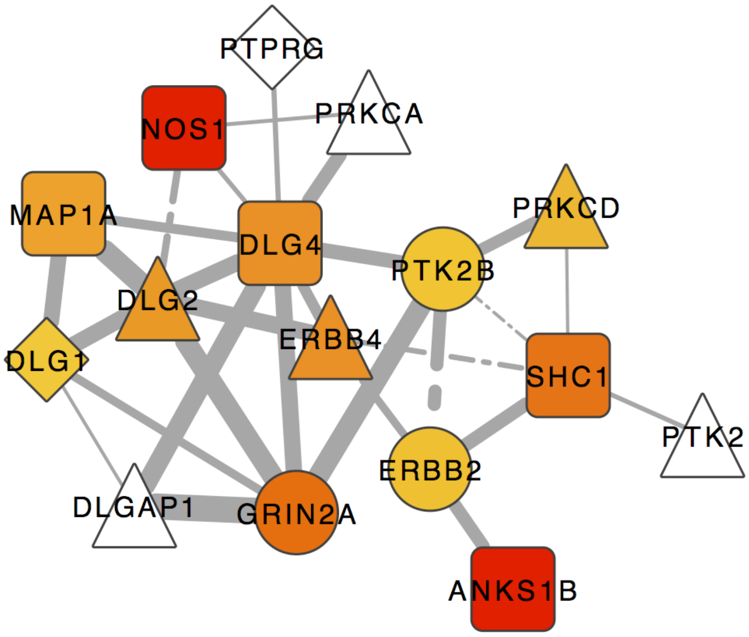

2. Top three significant modules identified when gene-level significance was set at $P_{node} < 0.05$

Module 1 (seed: MAPK8IP1_TIAM1)

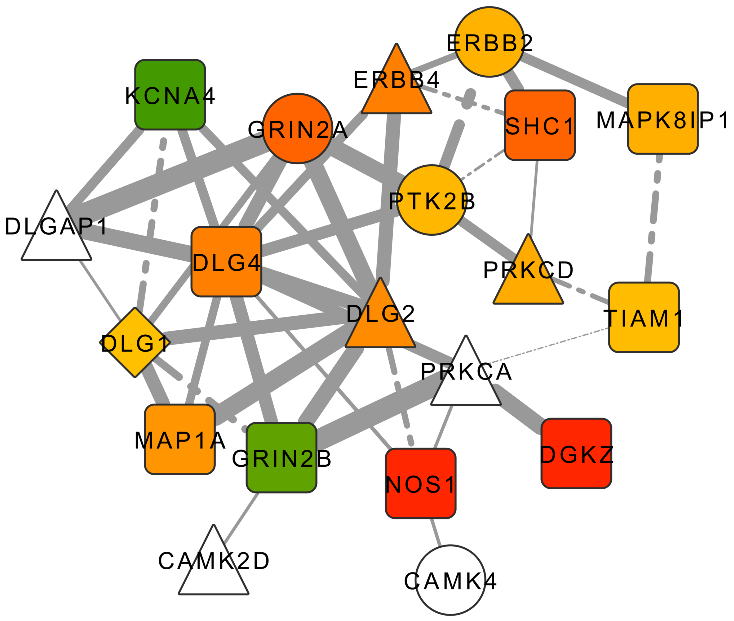

Module 2 (seed: IRS1_YWHAZ)

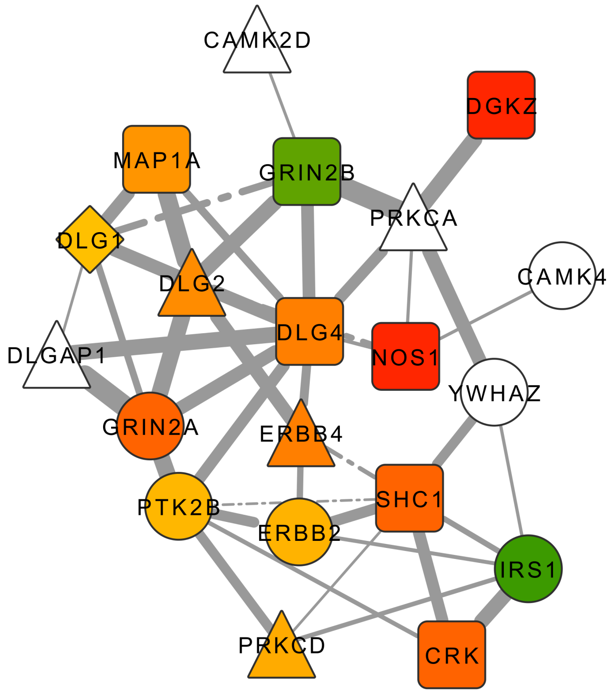

Module 3 (seed: AKT1_NCF1)

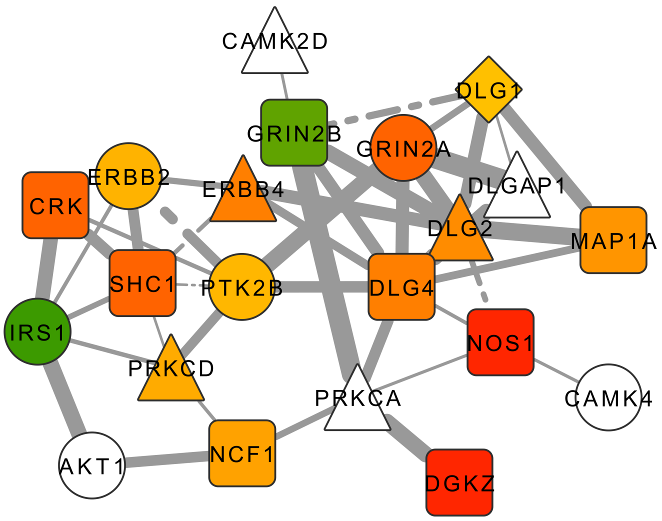

Figure S4 Regional plot of the gene loci identified from the PPI network, which show strong associations with schizophrenia risk, Plotted are the significance of association (–log10-transformed *P* values) and the recombination rate. SNPs are colored to reflect pairwise LD (r2) with the most significantly associated genotyped SNP. The most significant genotyped SNPs are marked in purple. The SNP association results are from PGC2 study.

1. *GRIN2A*

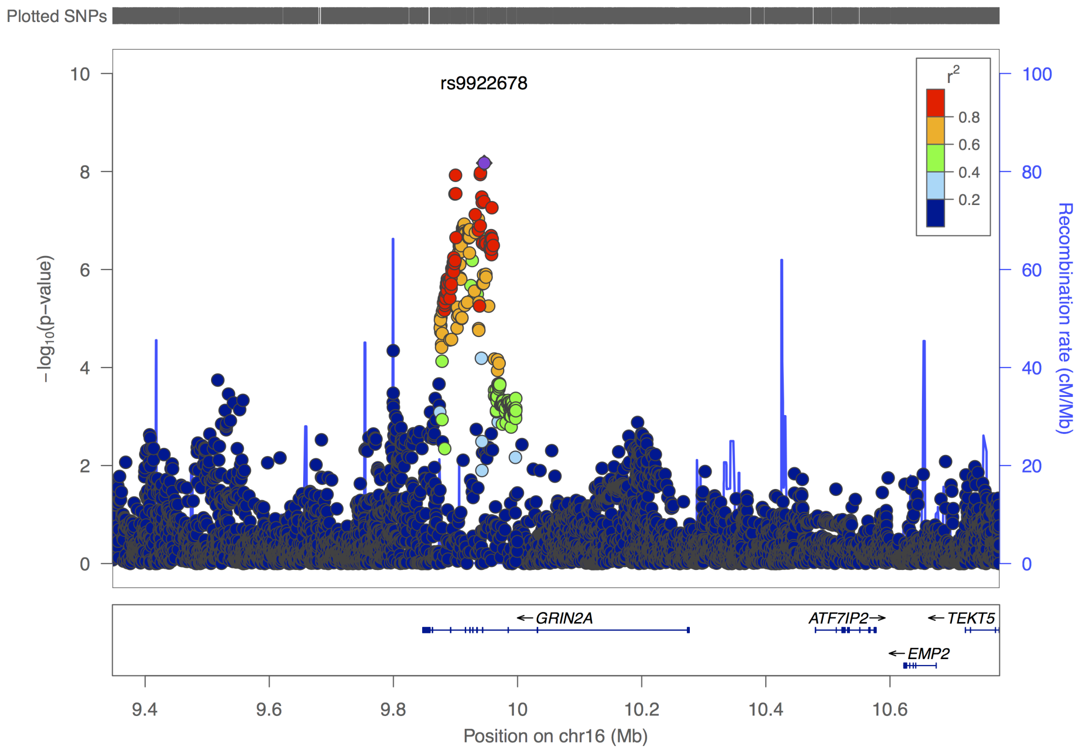

2. *GRIN2B*

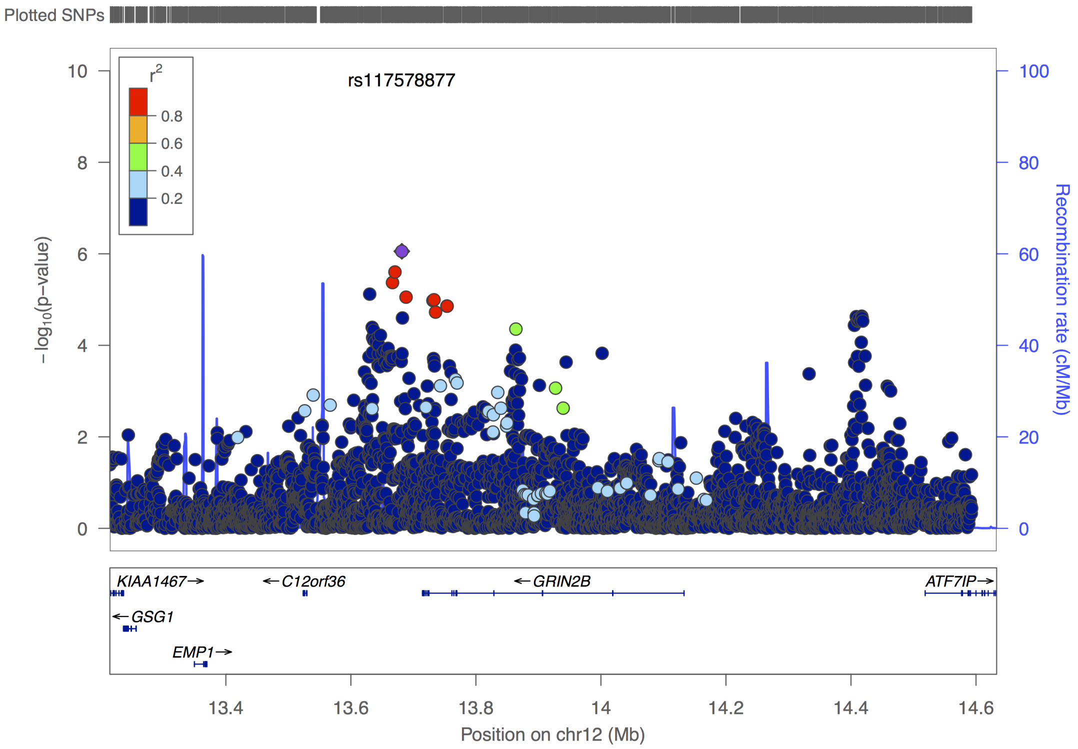

3. *DLG2*

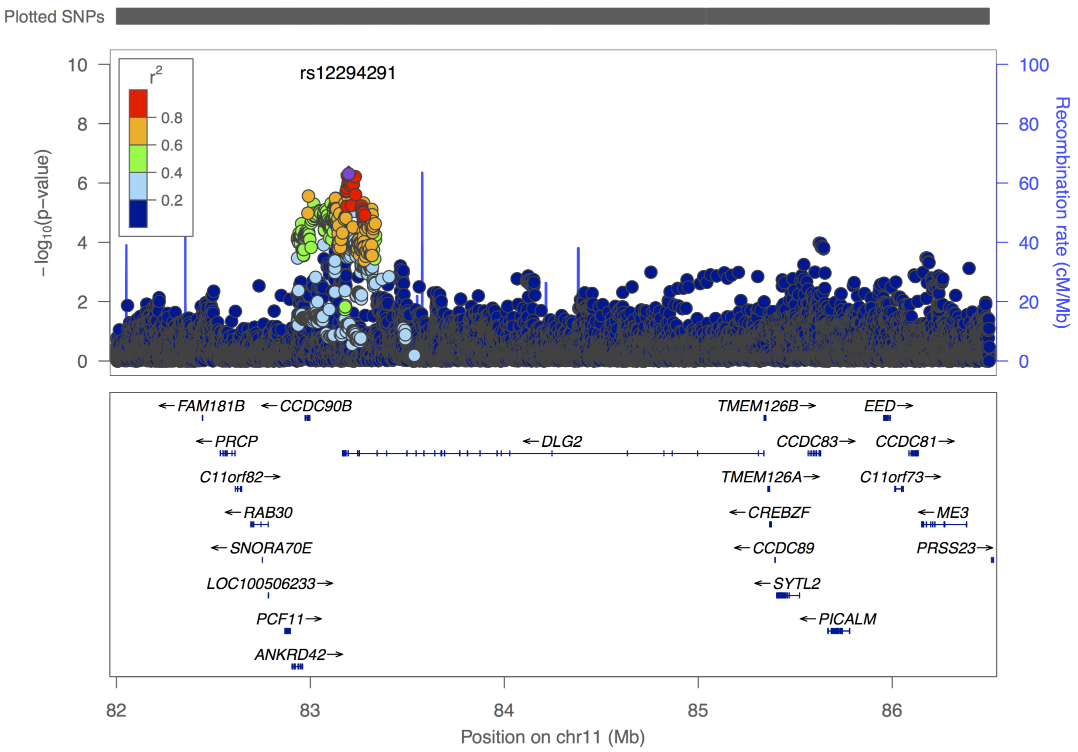

4. *DLG1*

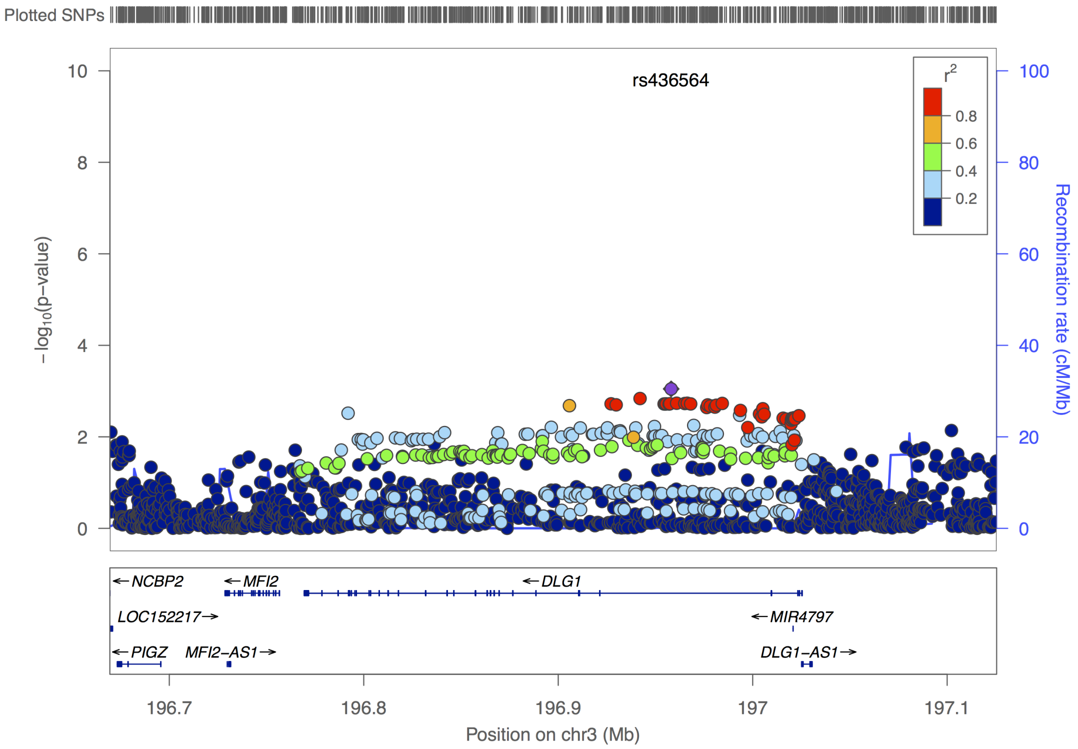

Plotted SNPs
rs436564
r2
0.8
0.6
0.4
0.2
−log10(p−value)
Recombination rate (cM/Mb)
NCBP2
MFI2
DLG1
LOC152217
MIR4797
PIGZ
MFI2−AS1
DLG1−AS1
196.7
196.8
196.9
197
197.1
Position on chr3 (Mb)

5. *DLG4*

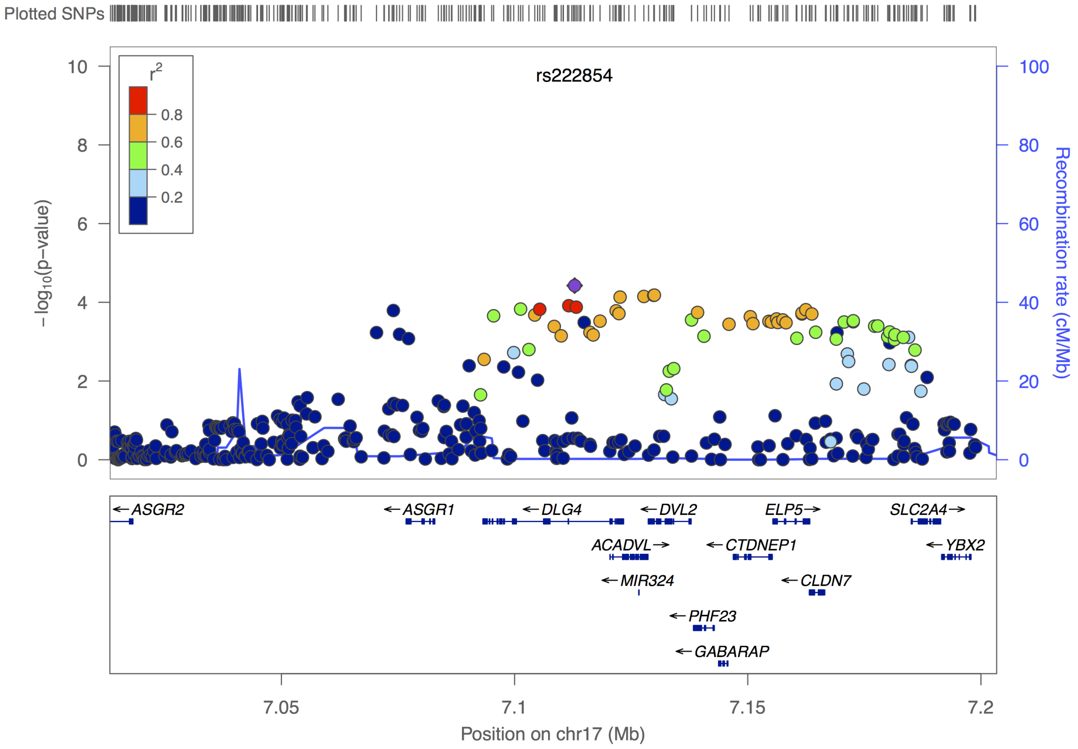

Plotted SNPs
rs222854
r2
0.8
0.6
0.4
0.2
−log10(p−value)
Recombination rate (cM/Mb)
ASGR2
ASGR1
DLG4
DVL2
ELP5
SLC2A4
ACADVL
CTDNEP1
YBX2
MIR324
CLDN7
PHF23
GABARAP
7.05
7.1
7.15
7.2
Position on chr17 (Mb)

6. *ATP2B2*

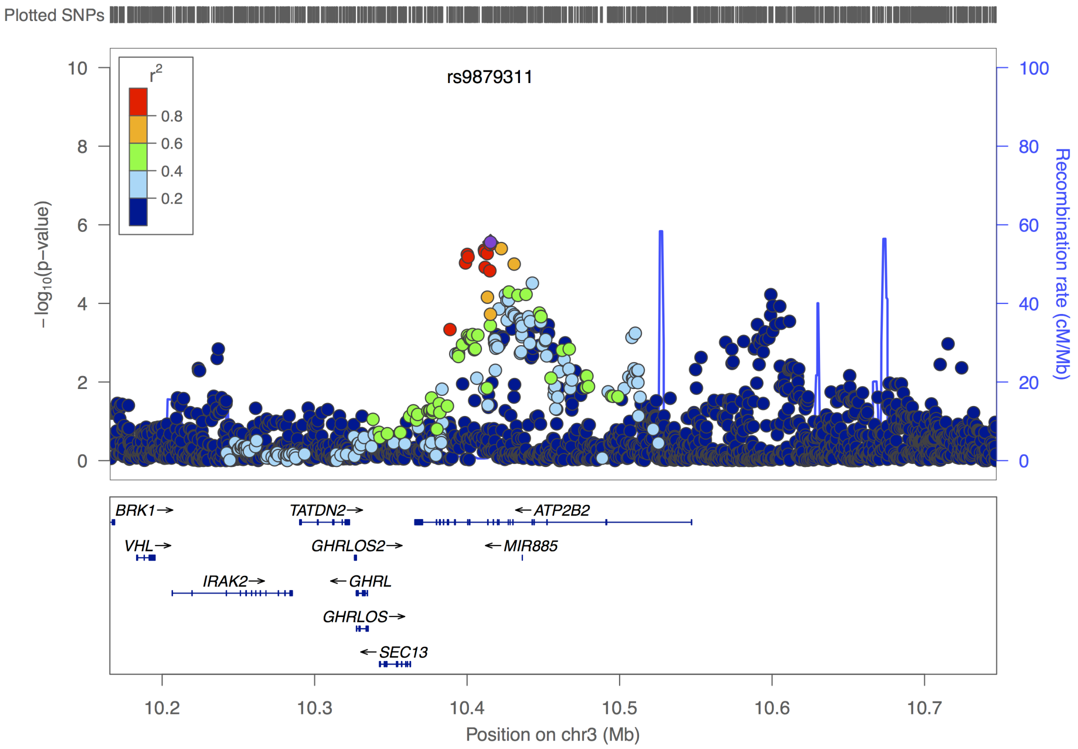

7. *NOS1*

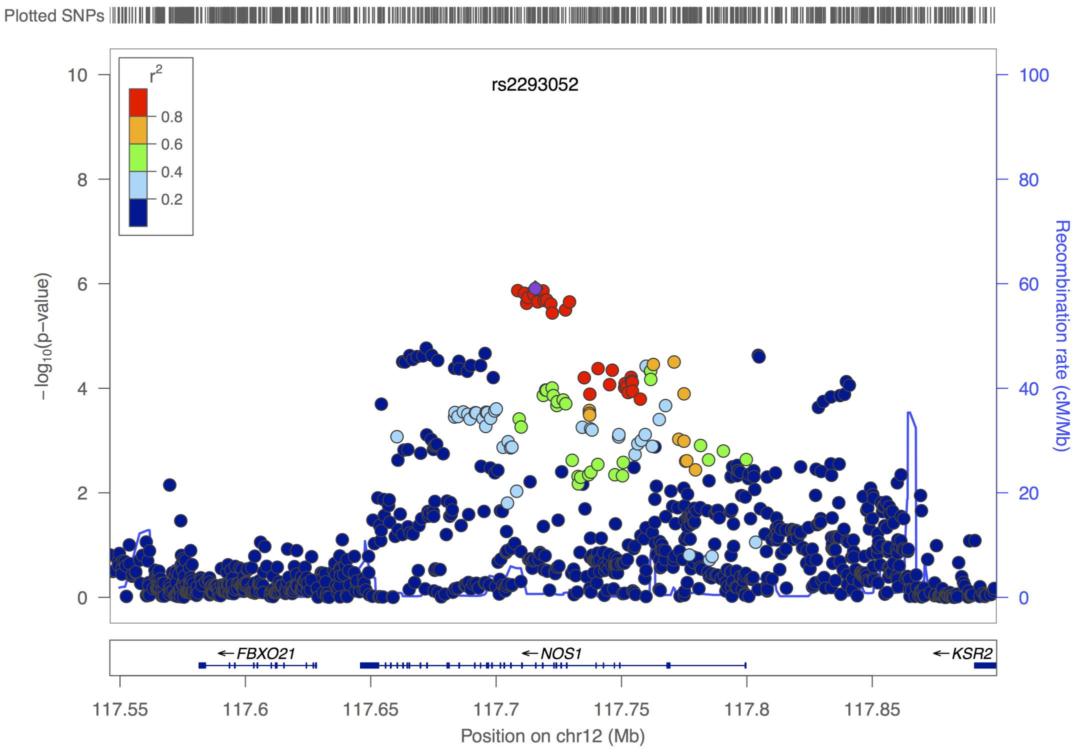

8. *ERBB4*

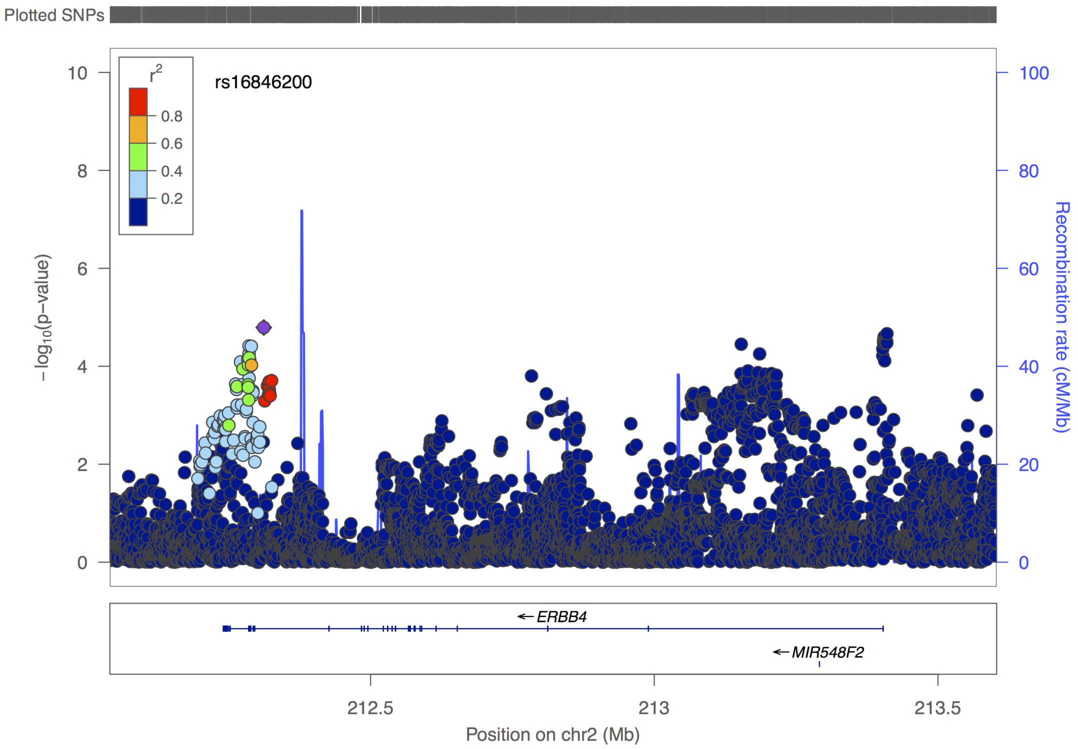

9. *ANSK1B*

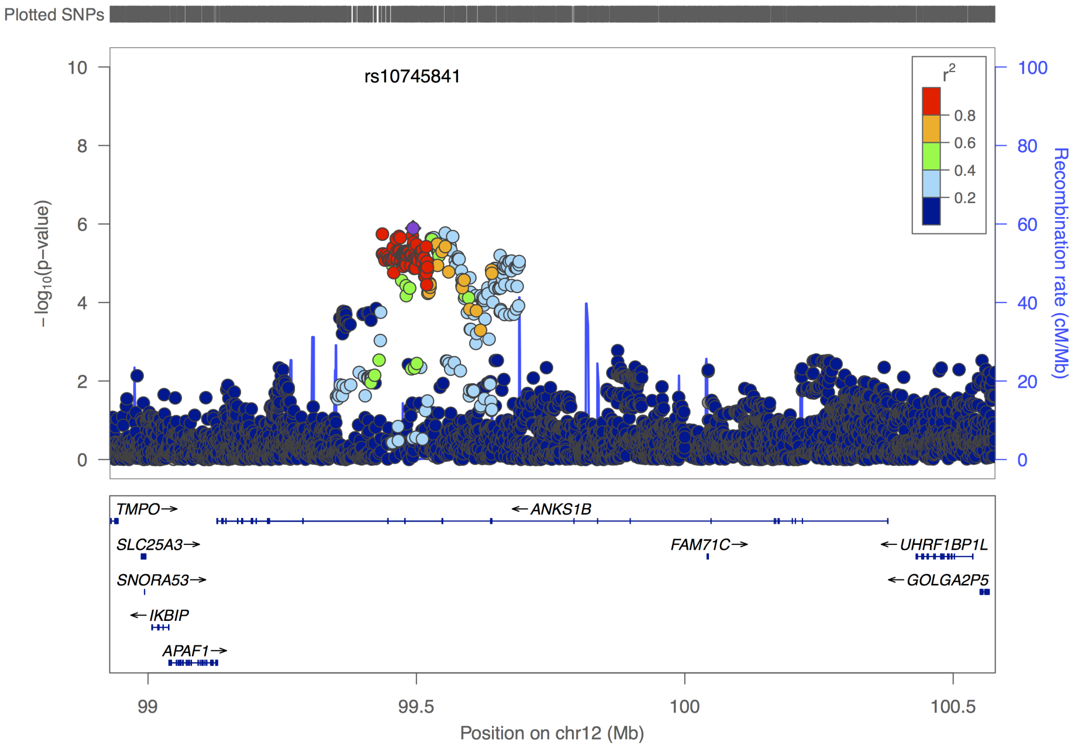

10. *CHUK*

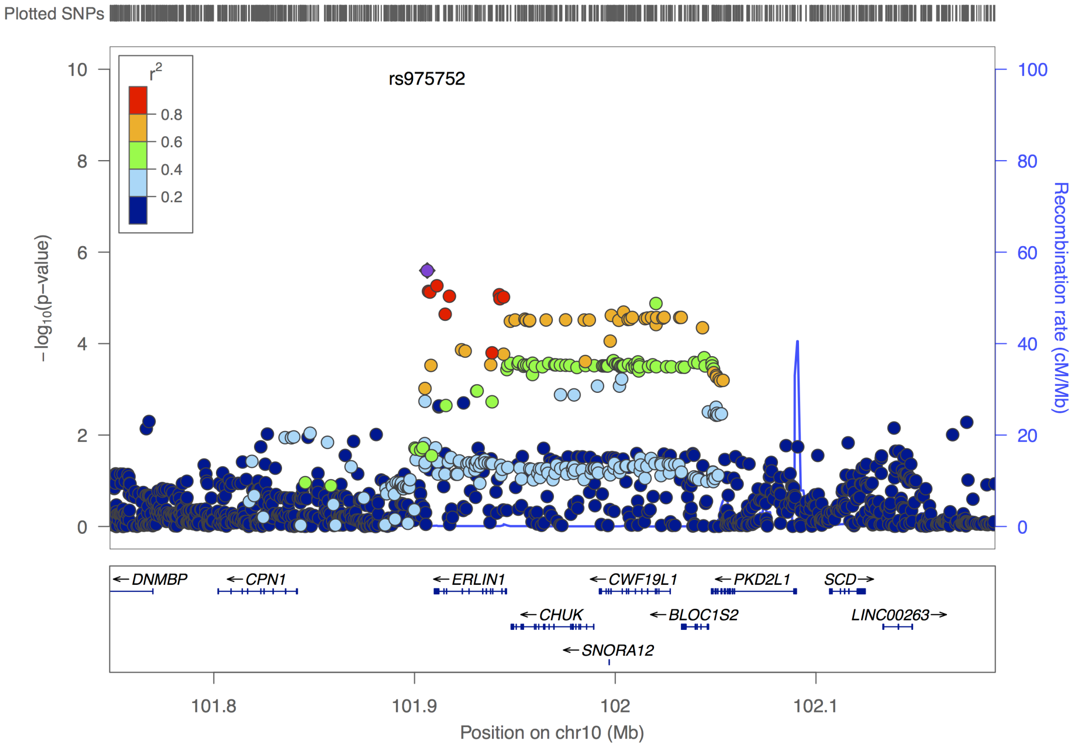

11. *CNTN2*

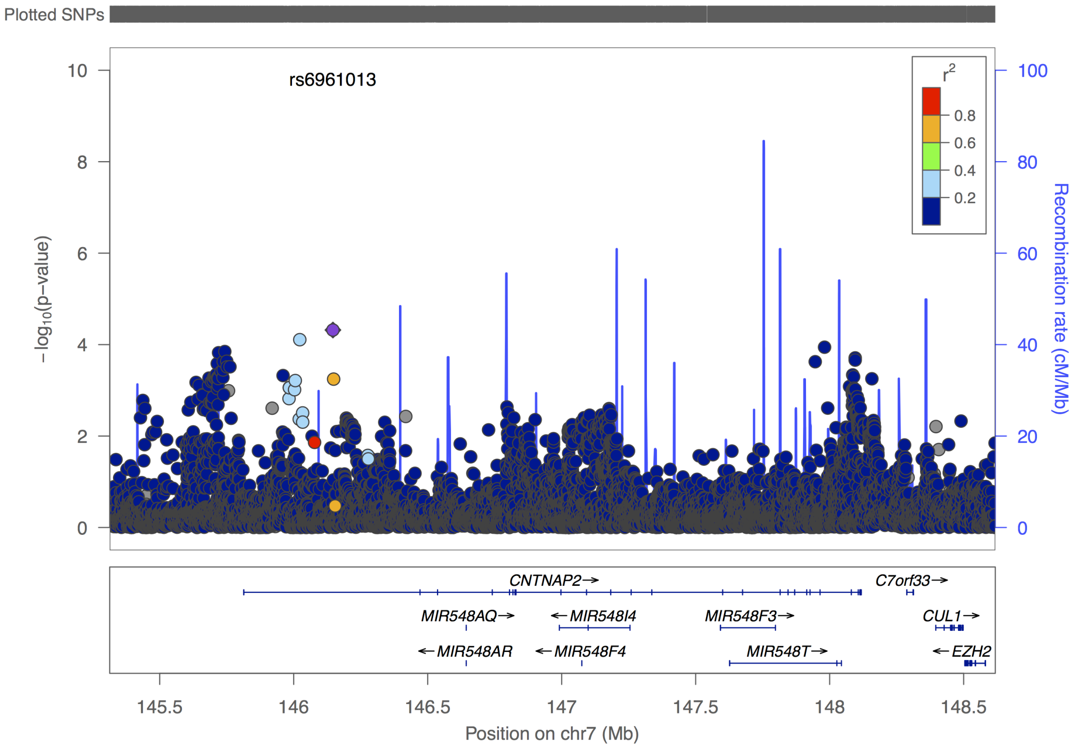

12. *CNTNAP2*

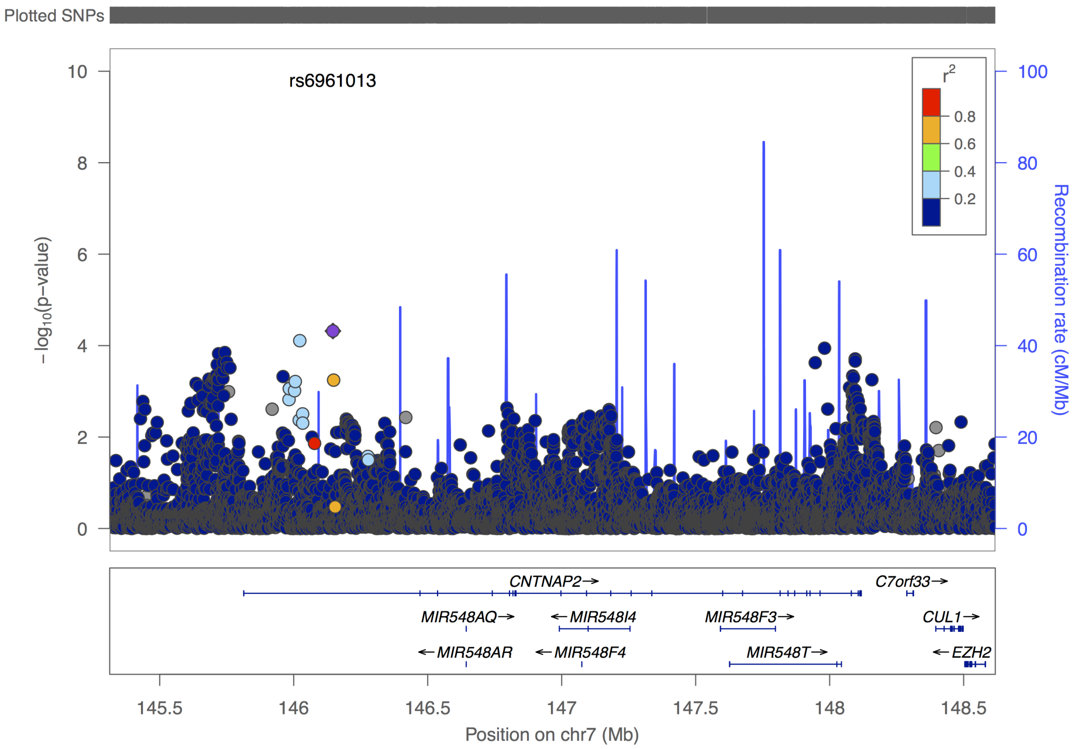

13. *CUL3*

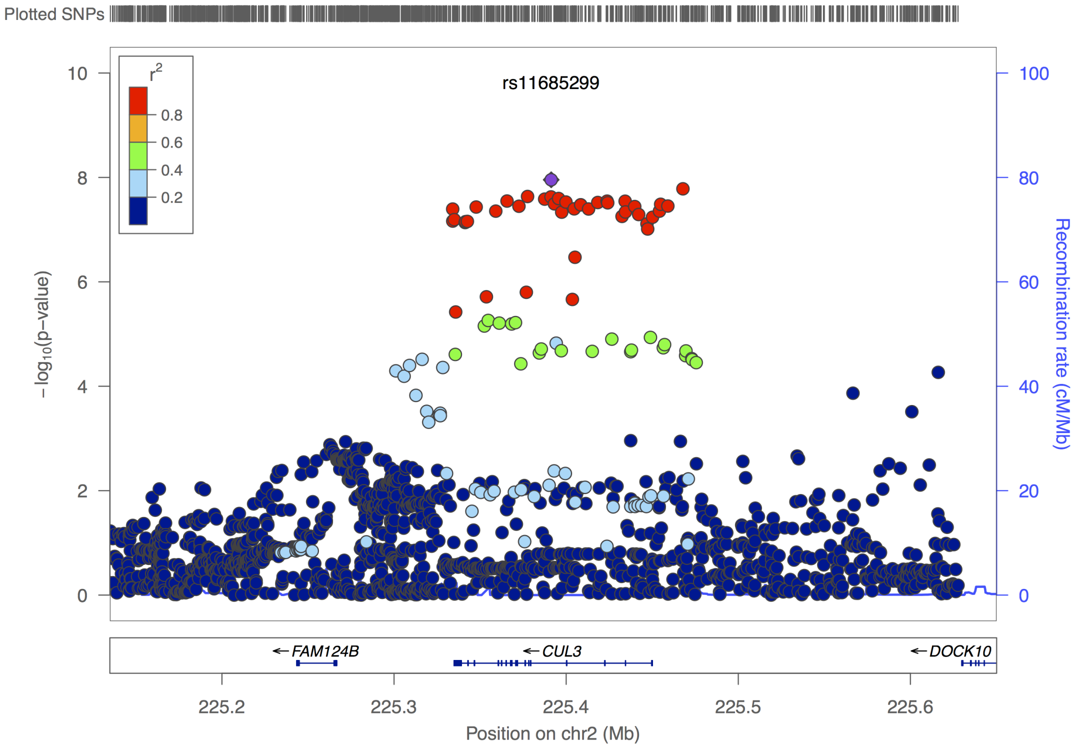

14. *CREB1*

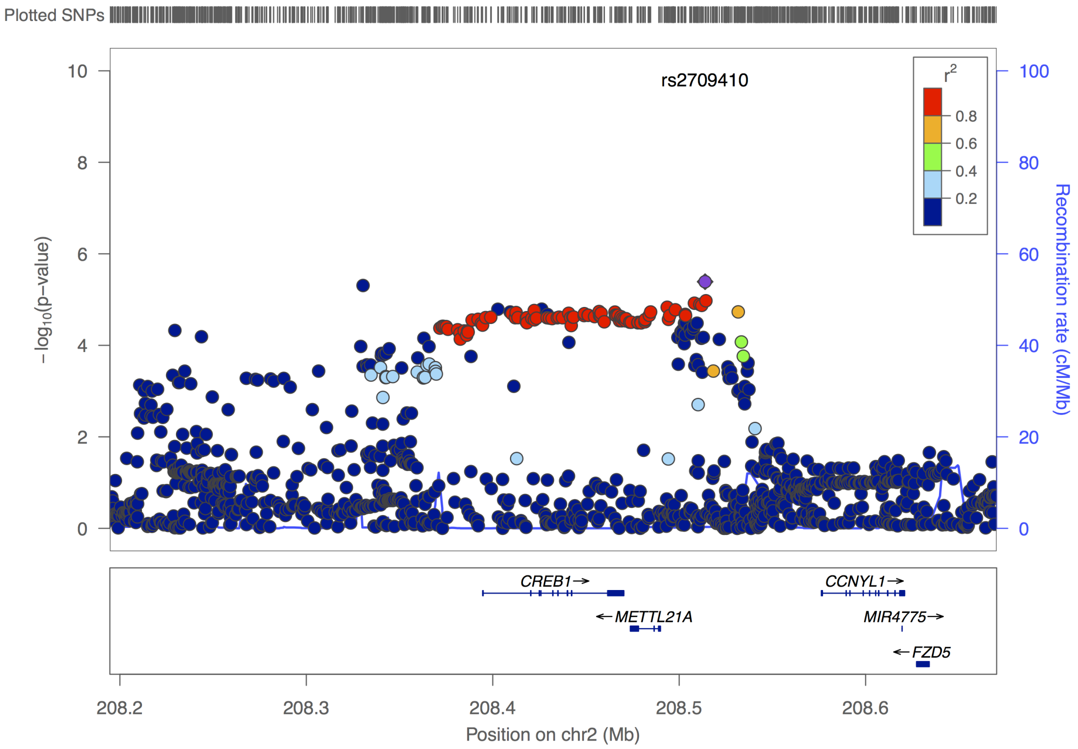

15. *CREB5*

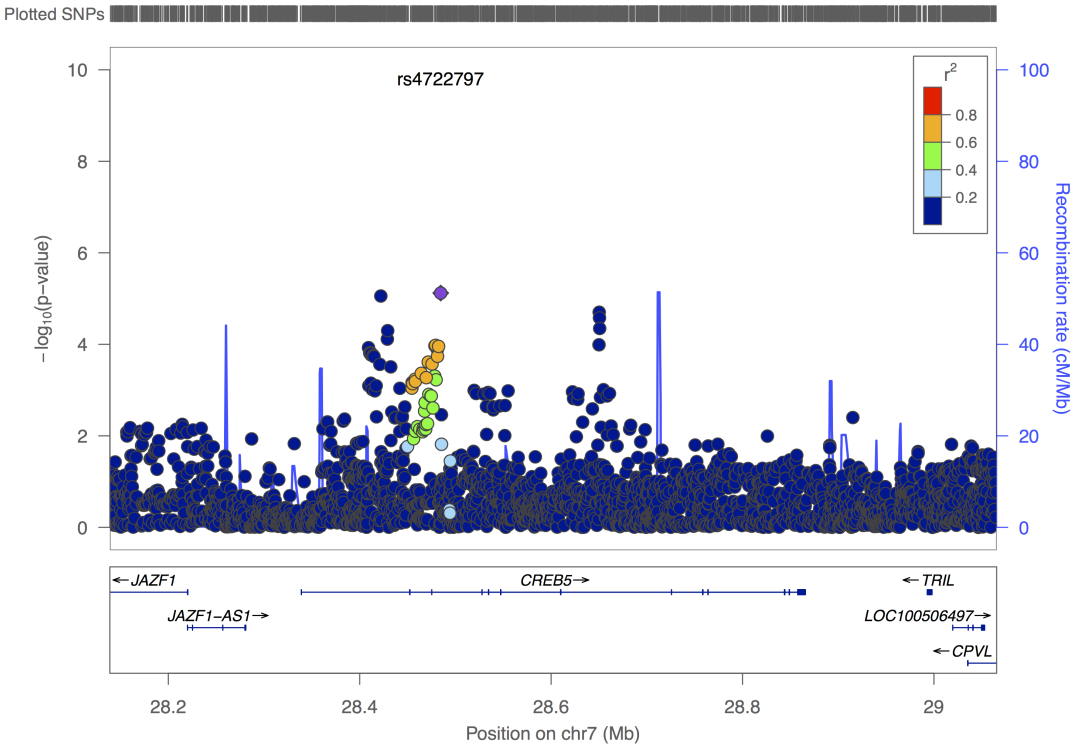

16. *EP300*

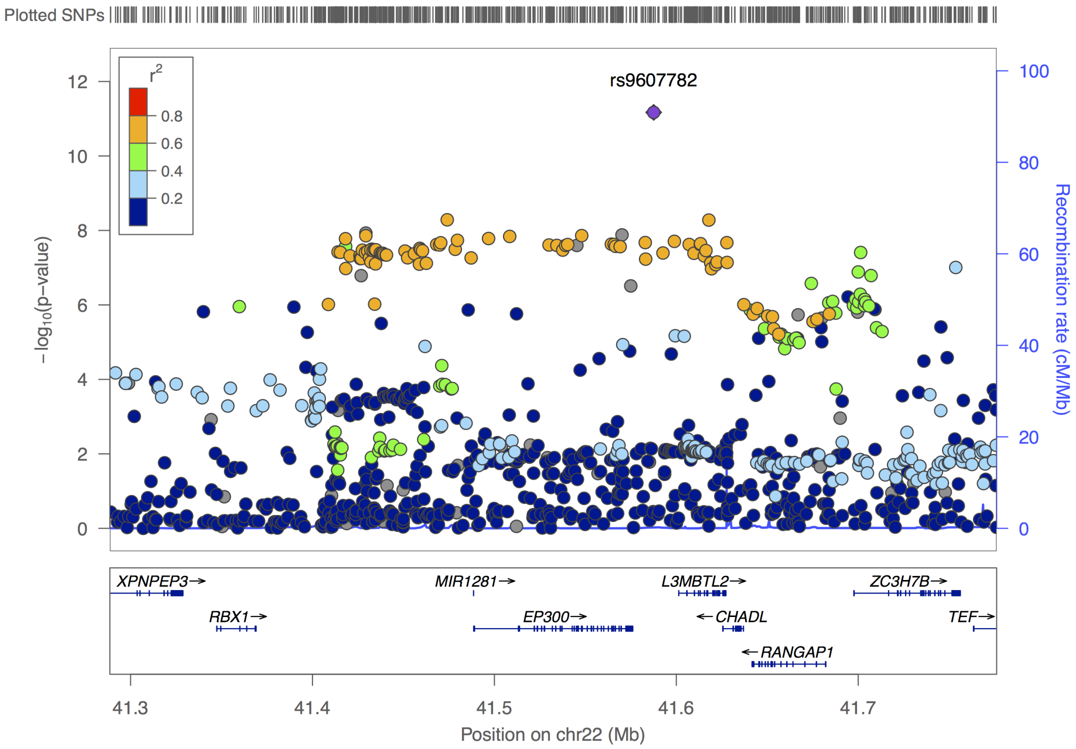

17. *GABBR2*

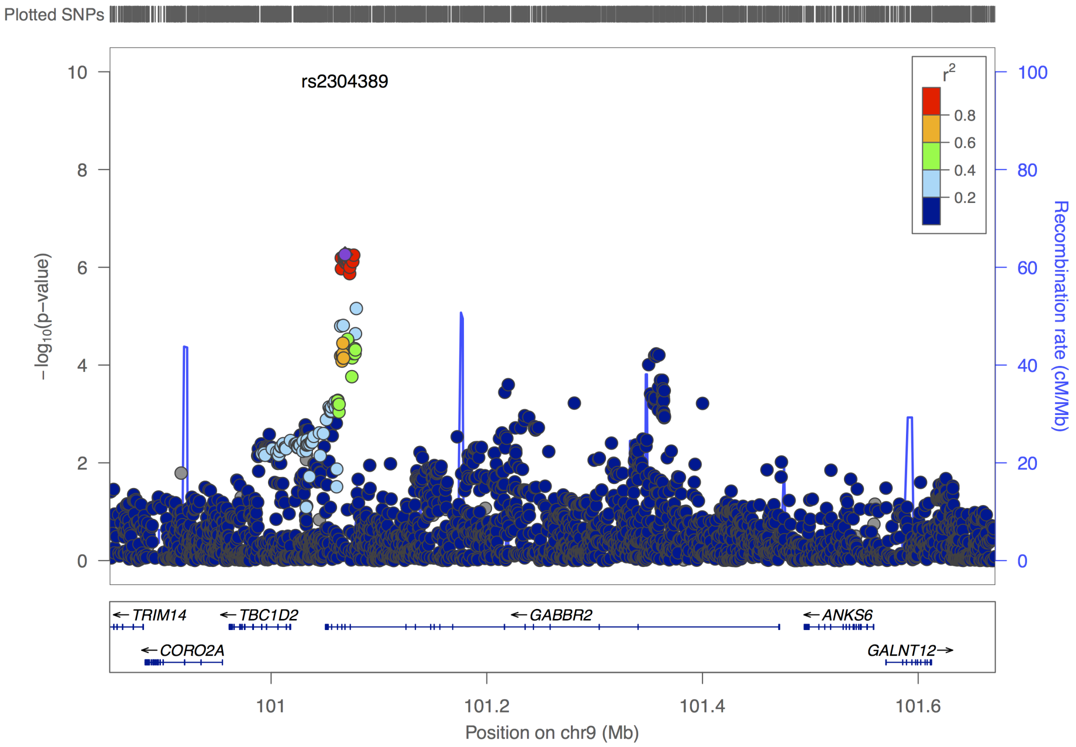

18. *GNA13*

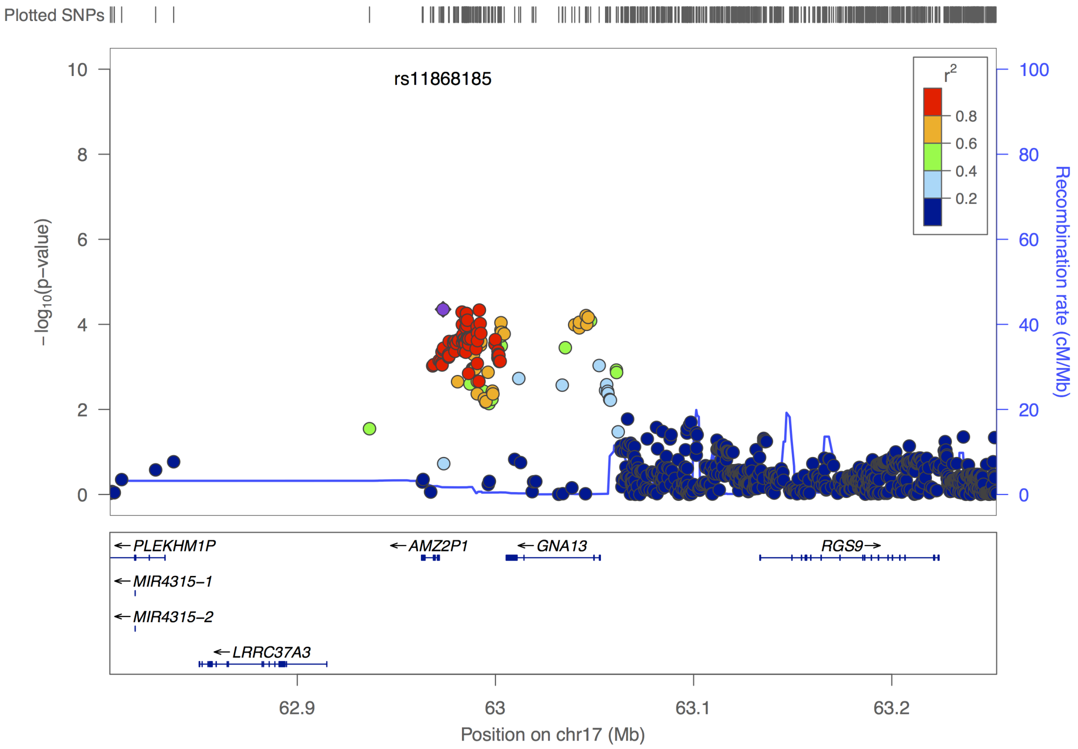

19. *NCOR2*

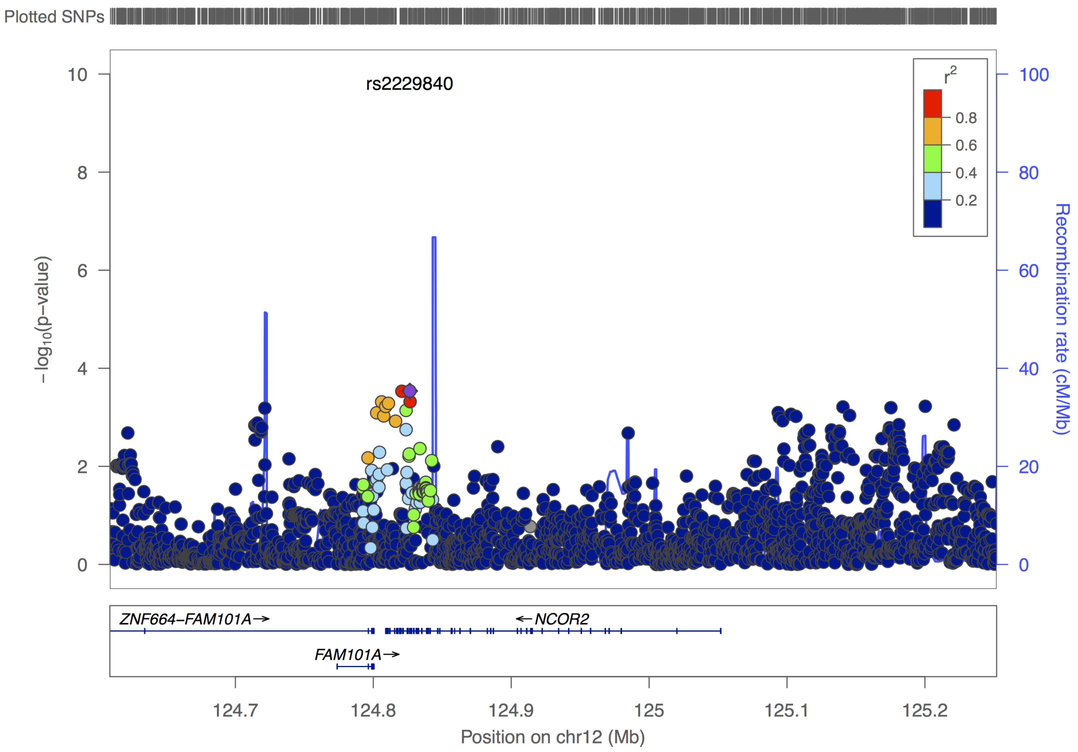

20. *NTRK3*

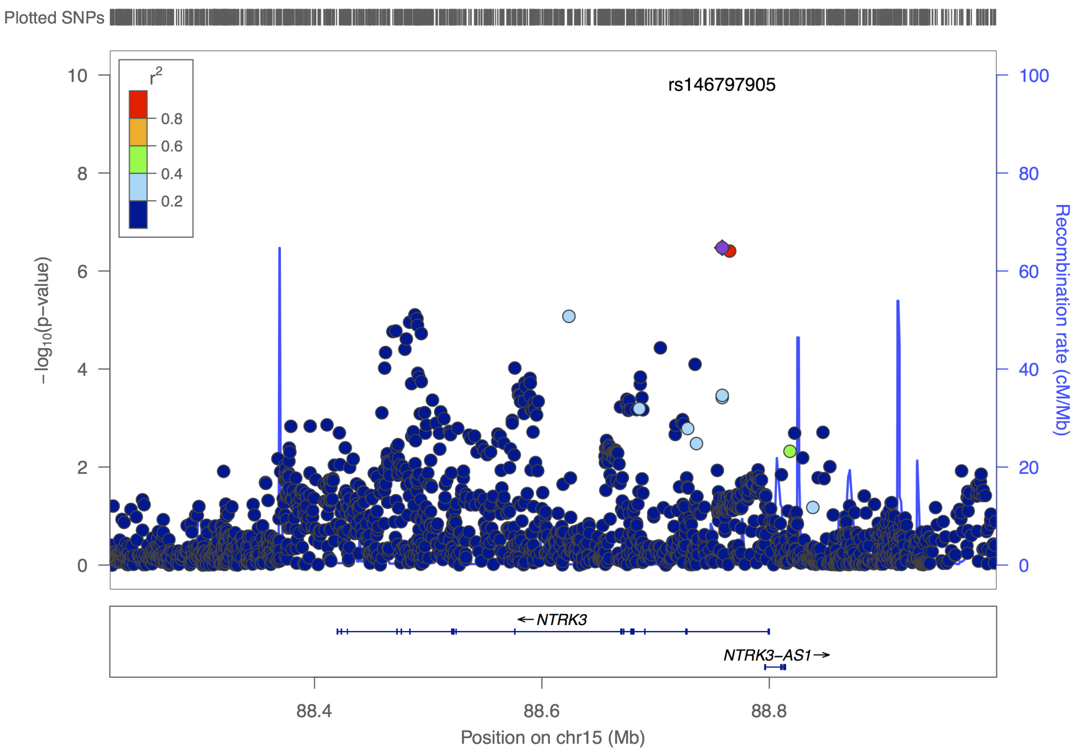

21. *NTRK3* (remove rs146797905)

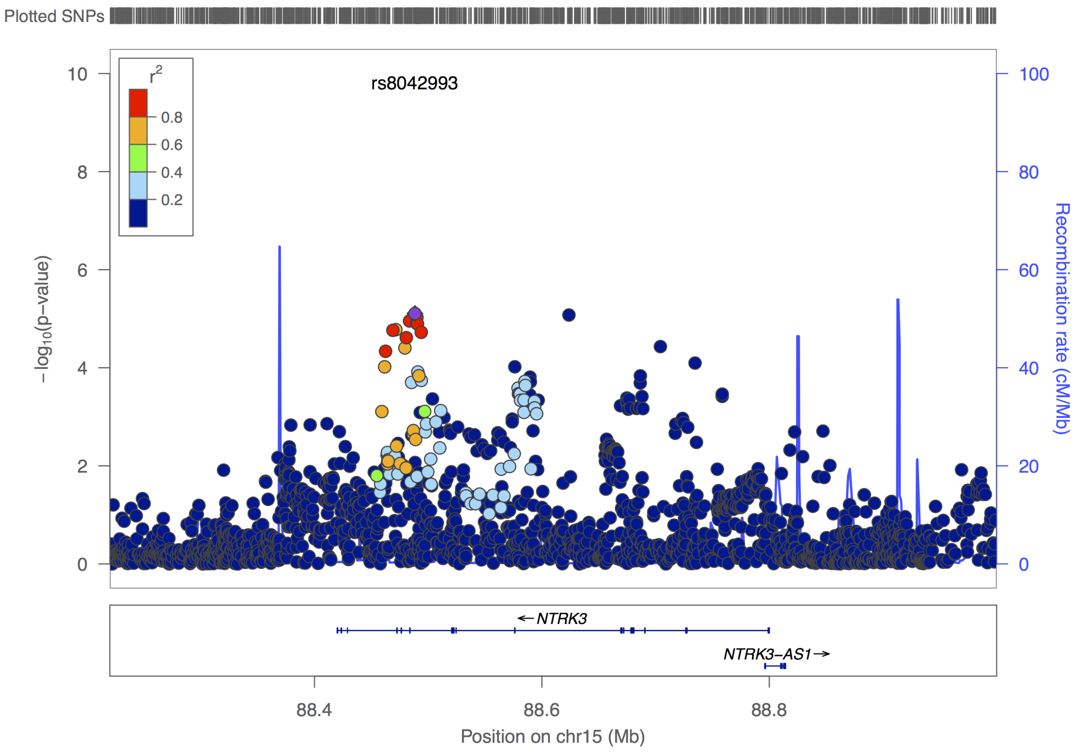

22. *PAK2*

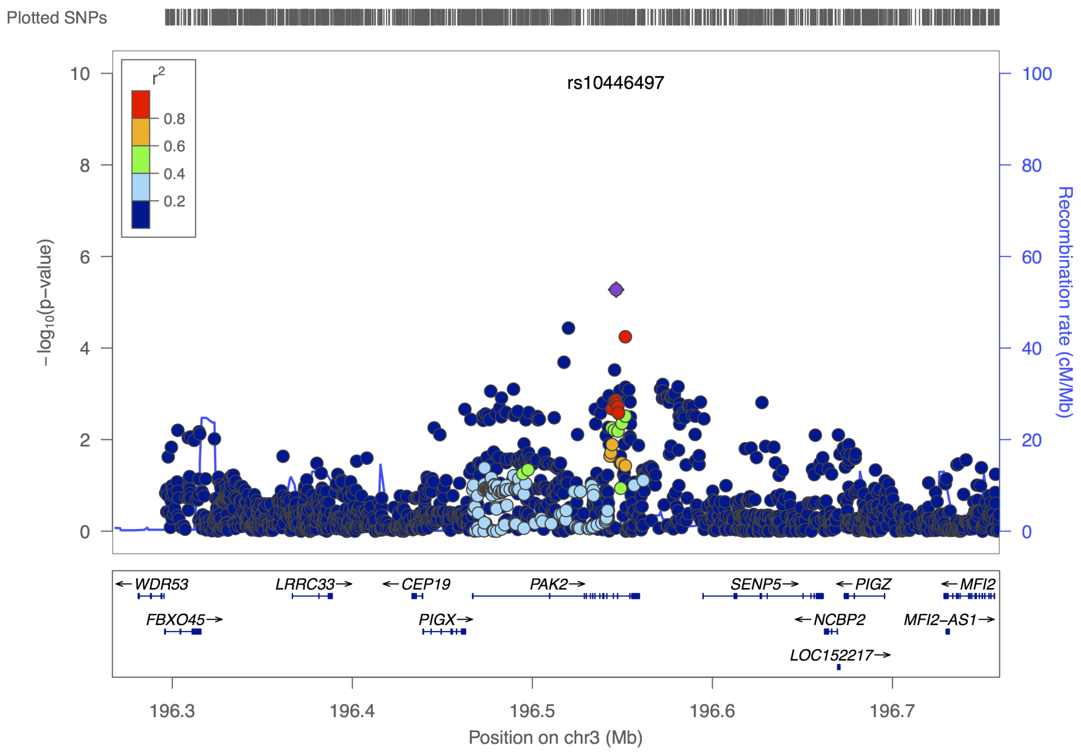

23. *PTK2*

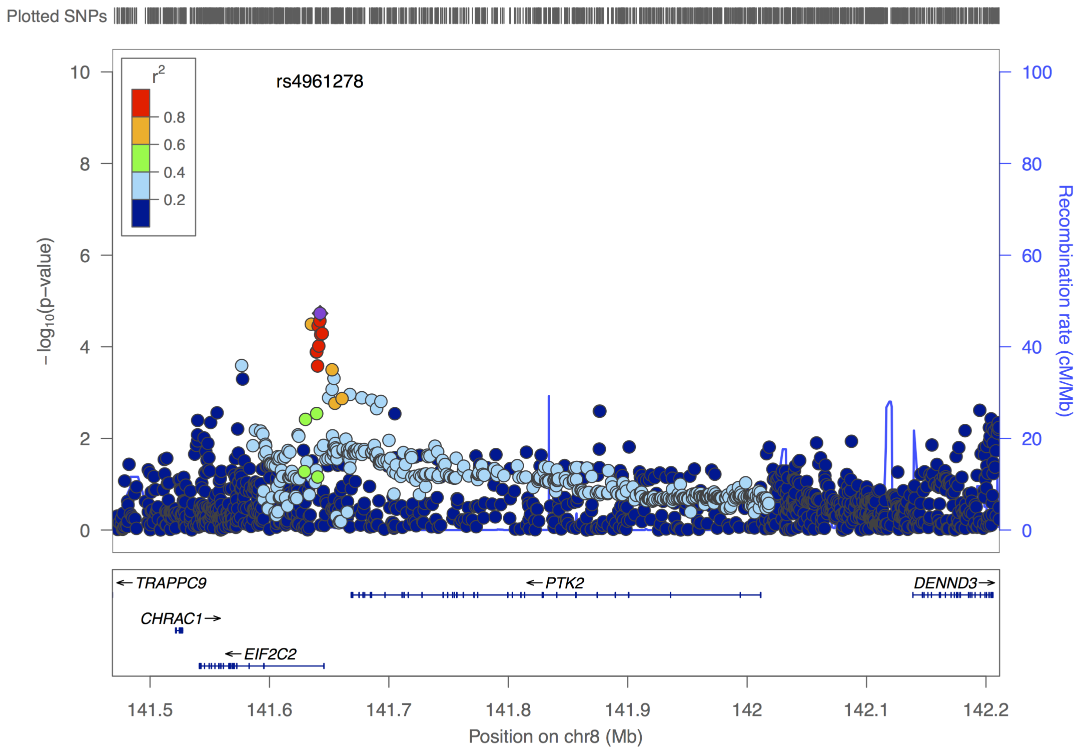

24. *PTK2B*

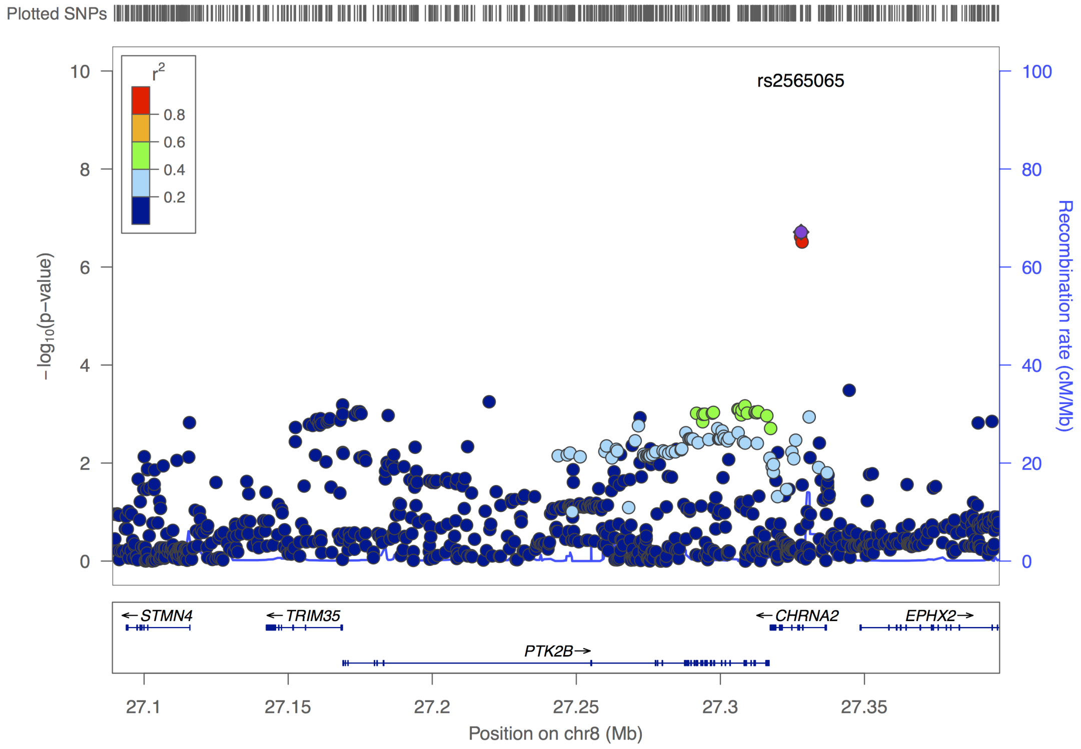

25. *PTN*

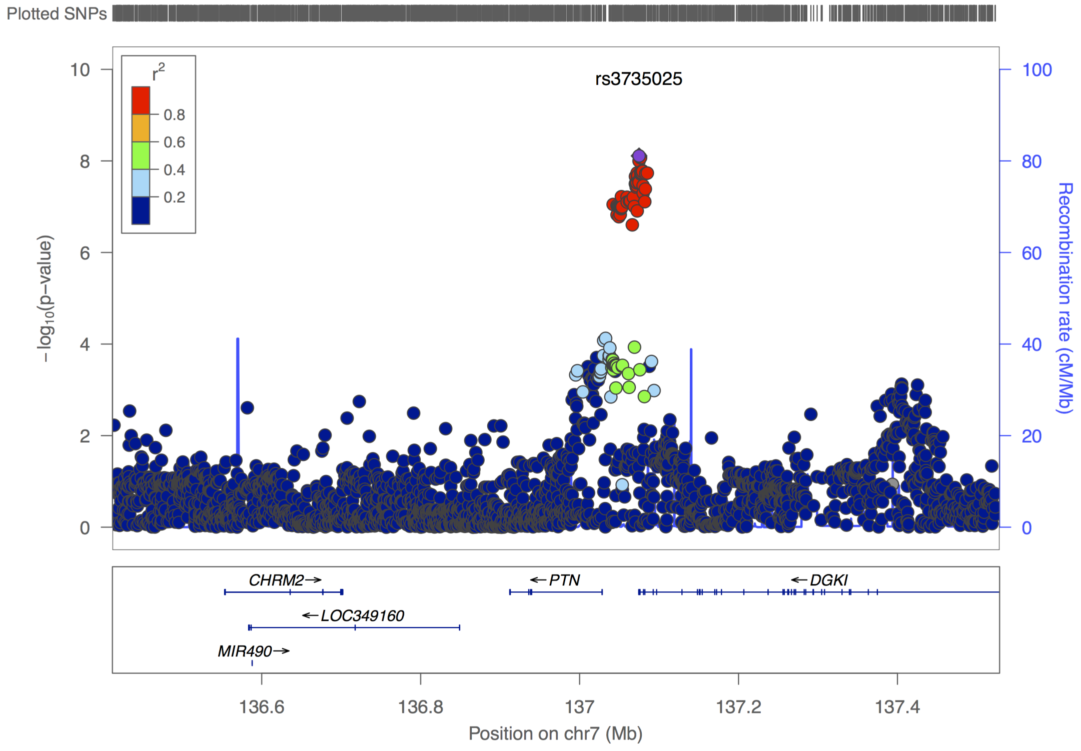

26. *PTPRF*

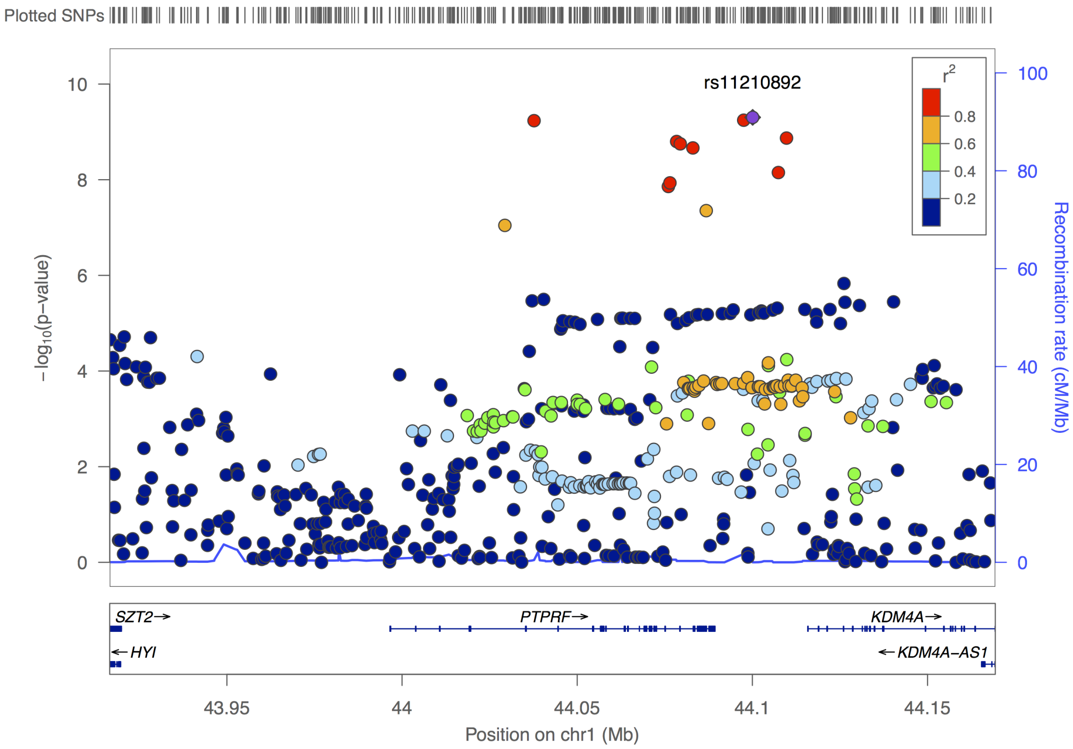

27. *STK4*

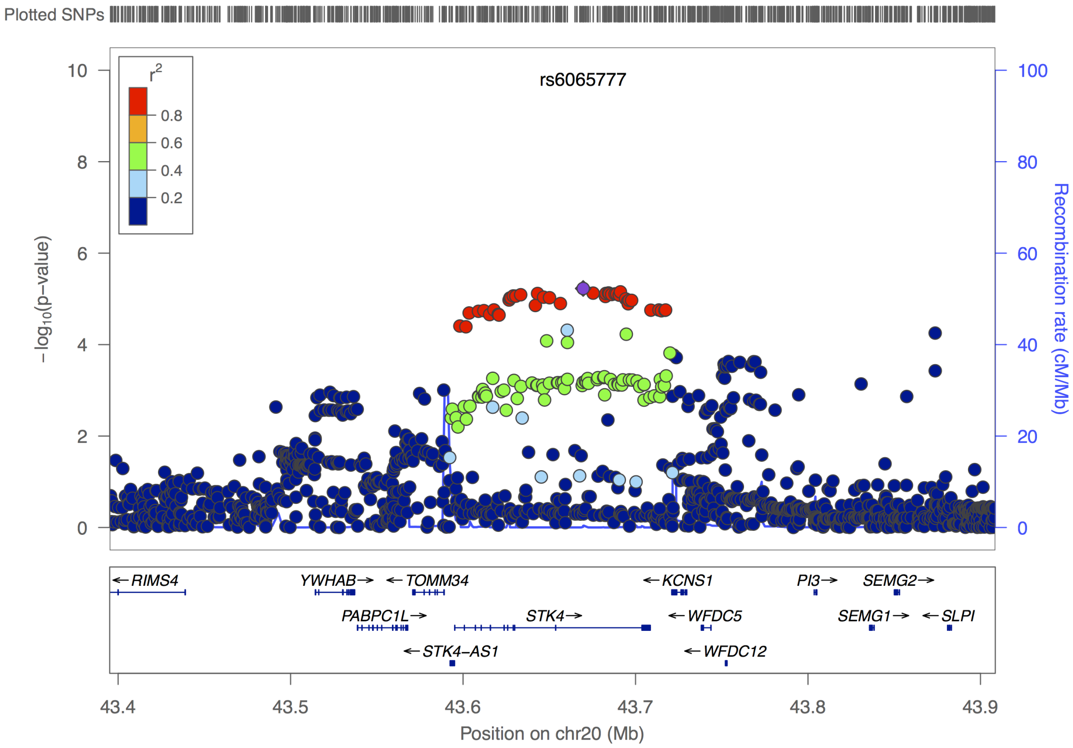

28. *TCF4*

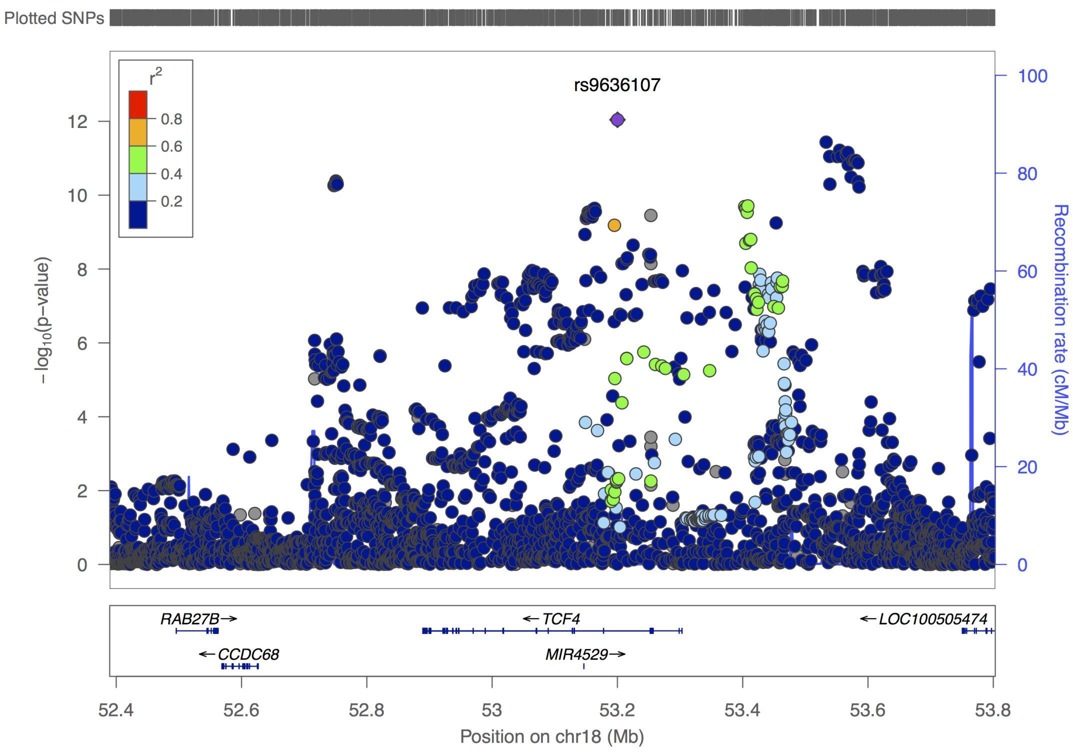

Plotted SNPs
rs9636107
r2
0.8
0.6
0.4
0.2
−log10(p−value)
Recombination rate (cM/Mb)
RAB27B
CCDC68
TCF4
MIR4529
LOC100505474
52.4
52.6
52.8
53
53.2
53.4
53.6
53.8
Position on chr18 (Mb)

Figure S5 Venn diagram summarizes the number of shared genes disrupted by DNMs in three exome sequencing studies of schizophrenia.

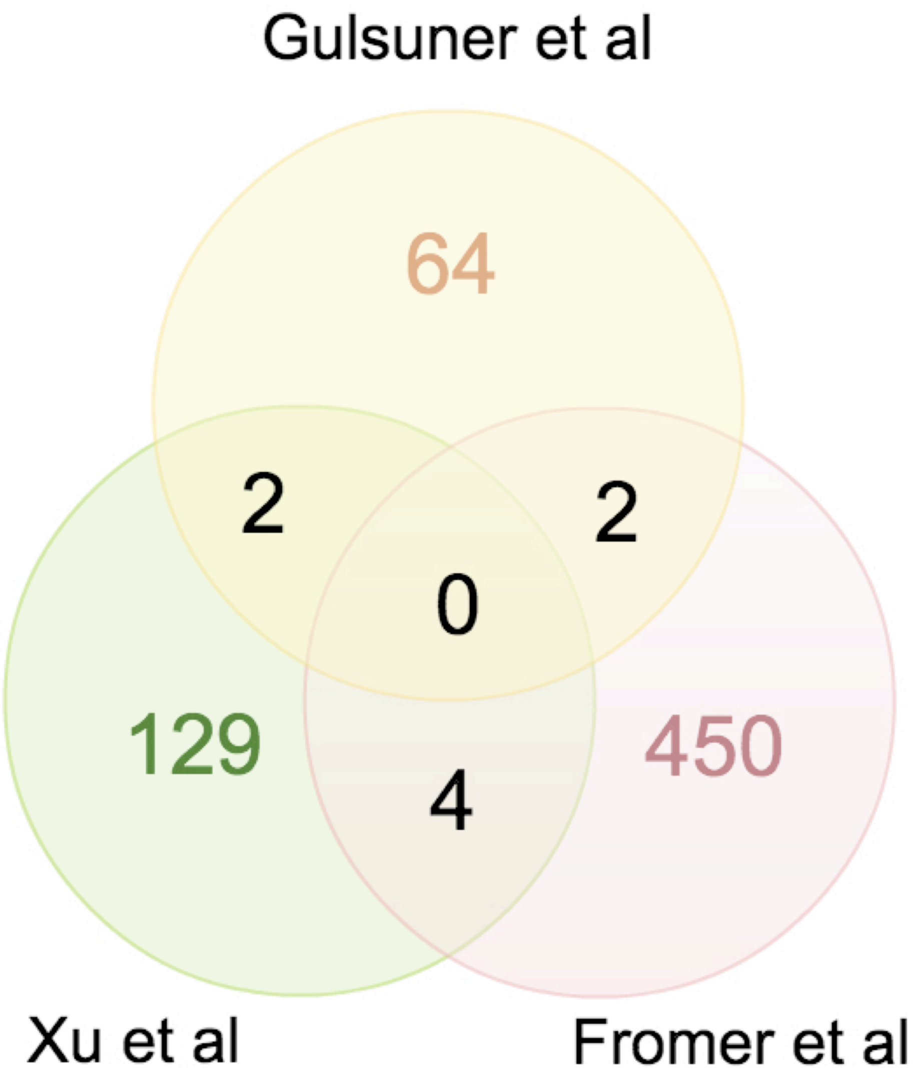

1. Qiao, D. *et al.* On the simultaneous association analysis of large genomic regions: a massive multi-locus association test. *Bioinformatics* **30**, 157-64 (2014).
2. Chuang, H.Y., Lee, E., Liu, Y.T., Lee, D. & Ideker, T. Network-based classification of breast cancer metastasis. *Mol Syst Biol* **3**, 140 (2007).
3. Jia, P., Zheng, S., Long, J., Zheng, W. & Zhao, Z. dmGWAS: dense module searching for genome-wide association studies in protein-protein interaction networks. *Bioinformatics* **27**, 95-102 (2011).
4. Ishizuka, K., Paek, M., Kamiya, A. & Sawa, A. A review of Disrupted-In-Schizophrenia-1 (DISC1): neurodevelopment, cognition, and mental conditions. *Biol Psychiatry* **59**, 1189-97 (2006).
5. Harrison, P.J. & Law, A.J. Neuregulin 1 and schizophrenia: genetics, gene expression, and neurobiology. *Biol Psychiatry* **60**, 132-40 (2006).
6. Clinton, S.M., Haroutunian, V., Davis, K.L. & Meador-Woodruff, J.H. Altered transcript expression of NMDA receptor-associated postsynaptic proteins in the thalamus of subjects with schizophrenia. *Am J Psychiatry* **160**, 1100-9 (2003).
7. Clinton, S.M., Haroutunian, V. & Meador-Woodruff, J.H. Up-regulation of NMDA receptor subunit and post-synaptic density protein expression in the thalamus of elderly patients with schizophrenia. *J Neurochem* **98**, 1114-25 (2006).
8. Walsh, T. *et al.* Rare structural variants disrupt multiple genes in neurodevelopmental pathways in schizophrenia. *Science* **320**, 539-43 (2008).
9. International Schizophrenia, C. Rare chromosomal deletions and duplications increase risk of schizophrenia. *Nature* **455**, 237-41 (2008).
10. Vrijenhoek, T. *et al.* Recurrent CNVs disrupt three candidate genes in schizophrenia patients. *Am J Hum Genet* **83**, 504-10 (2008).
11. Levinson, D.F. *et al.* Copy number variants in schizophrenia: confirmation of five previous findings and new evidence for 3q29 microdeletions and VIPR2 duplications. *Am J Psychiatry* **168**, 302-16 (2011).
12. Kirov, G. *et al.* De novo CNV analysis implicates specific abnormalities of postsynaptic signalling complexes in the pathogenesis of schizophrenia. *Mol Psychiatry* **17**, 142-53 (2012).
13. Xu, B. *et al.* Strong association of de novo copy number mutations with sporadic schizophrenia. *Nat Genet* **40**, 880-5 (2008).
14. Friedman, J.I. *et al.* CNTNAP2 gene dosage variation is associated with schizophrenia and epilepsy. *Mol Psychiatry* **13**, 261-6 (2008).
15. Vacic, V. *et al.* Duplications of the neuropeptide receptor gene VIPR2 confer significant risk for schizophrenia. *Nature* **471**, 499-503 (2011).
16. Mulle, J.G. *et al.* Microdeletions of 3q29 confer high risk for schizophrenia. *Am J Hum Genet* **87**, 229-36 (2010).
17. Glessner, J.T. *et al.* Strong synaptic transmission impact by copy number variations in schizophrenia. *Proc Natl Acad Sci U S A* **107**, 10584-9 (2010).
